# Repurposing Nilotinib as a Selective P38β Inhibitor in Hematopoietic Malignancies: Clinical Evidence and Mechanistic Insights

**DOI:** 10.33696/haematology.6.067

**Published:** 2025

**Authors:** Xu Hannah Zhang, Jack Hsiang, Sangkil Nam, Hongzhi Li, Yate-Ching Yuan, Xiwei Wu, Susan Hmwe, Roger Moore, Steven T. Rosen

**Affiliations:** 1 Department of Hematology & Hematopoietic Cell Transplantation, Beckman Research Institute, City of Hope, Duarte, CA, USA; 2 High Throughput Screening Core Shared research facility, Beckman Research Institute, City of Hope, Duarte, CA, USA; 3 Bioinformatics Core Shared Research Facilities, Beckman Research Institute, City of Hope, Duarte, CA, USA; 4 Integrative Genomics Core Shared Research Facilities, Beckman Research Institute, City of Hope, Duarte, CA, USA; 5 Department of System Clinical Trial Technology & Data Solutions Department, Beckman Research Institute, City of Hope, Duarte, CA, USA; 6 Integrated Mass Spectrometry Core Facility, Beckman Research Institute, City of Hope, Duarte, CA, USA

**Keywords:** Chronic myeloid leukemia, Clinical trials, Cutaneous T cell lymphoma, Nilotinib, Paclitaxel

## Abstract

**Background:**

Cutaneous T cell lymphoma (CTCL) is an incurable cancer characterized by elevated p38β and p38γ and downregulated tumor-suppressive p38α.

**Objectives:**

We aimed to identify selective p38β inhibitors and investigate their mechanisms and therapeutic implications in hematologic malignancies.

**Methods:**

A high-throughput screen of Food and Drug Administration (FDA)-approved compounds was conducted to identify p38β inhibitors. *In vitro* kinase assays, Western blots, scRNA-seq, synergy tests, and mass spectrometry were used. Clinical trial and public datasets were analyzed.

**Results:**

Nilotinib was identified as a selective p38β inhibitor (~25-fold over p38α). Its inhibition of p38β led to compensatory activation of p38γ, driving Epithelial-to-Mesenchymal Transition (EMT) and potential toxicities. Combining Nilotinib with p38γ inhibitors (e.g., CSH71 or Paclitaxel) produced synergistic anti-tumor effects in CTCL and leukemia cells. Clinical and FDA Adverse Event Reporting System (FAERS) data revealed higher toxicity rates for Nilotinib in Ph^+^ CML and off-target effects in non-CML cancers.

**Conclusion:**

Nilotinib selectively inhibits p38β but activates p38γ, potentially reducing therapeutic selectivity. Combining it with p38γ inhibitors may enhance efficacy and reduce toxicity across hematologic malignancies.

## Introduction

The p38 kinase family comprises four isoforms—p38α(MAPK14), p38β(MAPK11), p38γ(MAPK12), and p38δ(MAPK13)—each playing a unique role in cellular processes including inflammatory responses and cell differentiation. These isoforms have garnered attention in clinical research, particularly in the context of cancer, due to their involvement in tumor progression and response to stress. Among them, the role of p38β role in Cutaneous T cell lymphoma (CTCL), an incurable cancer, is of particular interest. Our previous studies have highlighted a significant increase in p38β gene expression in CTCL patient samples as compared to normal CD4^+^ T cells [1], suggesting it may be a promising therapeutic target.

A challenge in targeting the p38 pathway for cancer treatment lies in the structural similarity between p38α and p38β. The isoforms share approximately 80% of their amino acids and are about 75% structurally similar [2,3]. Their functionalities, however, are likely to be quite different. p38α is recognized as a tumor suppressor [2–5] and is essential for the survival of many cell types. In contrast, p38β levels increase in T malignancy CTCL. The precise role of p38β in carcinogenesis and progression of cancer, however, is still unclear.

Clinical trials involving non-selective p38 inhibitors that target both p38α and p38β have been hampered by adverse effects and drug resistance. Non-selective p38 inhibitors target both p38α and p38β, leading to adverse effects such as rash, fatigue, nausea, constipation, pruritus, and vomiting, as seen in the first Phase I clinical trial with the oral p38 MAPK inhibitor Ralimetinib (LY2228820 Dimesylate) [6], and in this trial, none of the 89 patients with advanced cancer achieved a complete or partial response. It is likely due to Ralimetinib’s targeting of p38α, which is recognized as a tumor suppressor and is essential for the survival of many cell types [2–5].

This underscores the necessity of developing p38β-specific inhibitors for effective CTCL treatments. In our quest for effective treatments for CTCL, we screened a library of Food and Drug Administration (FDA)-approved drugs to identify p38β-specific inhibitors to investigate the impacts of selected drugs in the clinical trials and CTCL cells. In addition, future p38β-targeted therapies should be rigorously evaluated and optimized to minimize pathway-related toxicities and overcome the limitations observed with non-selective p38 inhibition.

## Results

### Identification of Nilotinib as a potent p38β inhibitor

Given that p38β expression is elevated in CTCL cells, it is a promising therapeutic target. We conducted high-throughput screening of 1,443 FDA-approved compounds to identify specific inhibitors of p38β (Figure 1A). Using an *in vitro* p38β kinase assay, we identified several inhibitors, focusing on Linifanib, Ponatinib, and Nilotinib for further analysis (Figure 1B). To assess the specificity of these compounds against p38β kinase, we also determined the half-maximal inhibitory concentration (IC_50_) for p38α, p38γ, and p38δ, respectively (Table 1).

We found that Nilotinib is the most potent p38β inhibitor among four p38 isoforms, with an IC_50_ value of 0.014 μM, showing it is 25-fold more potent against p38β than p38α. For comparison, we included the FDA orphan drug Flavopiridol, previously identified as a pan p38 isoform inhibitor [7]. Nilotinib inhibited p38γ least effectively (IC_50_>10 μM) and had an IC_50_ over 4.45 μM for p38δ (Figure 1C).

Nilotinib, an FDA-approved drug for treating imatinib-resistant Chronic Myelogenous Leukemia (CML) [8–10], has been thoroughly studied and been found to bind p38β which was validated by a curated X-ray crystallography study [PDB: 3GP0, DOI: 10.2210/pdb3GP0/pdb]. Based on this structural data, we applied our All-Around Docking (AAD) method [11] to predict the optimal binding site of Nilotinib on p38β. The docking pose revealed three key hydrogen bonds formed with residues E71, M109, and D168 (Figure 1D, left). The analysis confirmed that Nilotinib, along with five other tyrosine kinase inhibitors (TKIs), binds within the ATP-binding pocket of p38β (Figure 1D, right).

### Inhibition of p38β induces classical dual phosphorylation of p38γ

To understand the functional consequences of p38β inhibition by Nilotinib in CTCL cells, we performed Western blot analysis and found that Nilotinib treatment induces dual phosphorylation of p38γ, a pro-oncogenic isoform in its active state. In Hut78 cells, Nilotinib treatment led to the appearance of an additional higher molecular weight band (>40 kDa), consistent with the dually phosphorylated form of p38γ (Figure 2A, shCtr Hut78 cell panel).

To confirm the identity of the top band, we employed a lentiviral shRNA system to knock down p38γ. The band disappeared in p38γ-silenced cells (shp38γ Hut78 cell panel), validating it as phosphorylated p38γ (blue arrow, Figure 2A). Notably, due to its slightly larger size (367 amino acids), p38γ migrates higher than other p38 isoforms (~360 amino acids, orange arrow in Figure 2A), and its phosphorylated form is clearly resolved from non-phosphorylated species on SDS-PAGE gels which was then detected using a phospho-p38 MAPK (Thr180/Tyr182) antibody (CST #4511). In addition, similar higher-molecular-weight phospho-p38γ bands have been observed in other p38β inhibitors SB203580 in myoblast, a progenitor cell for muscle cells [12], although the higher-molecular-weight bands were not further characterized in those studies.

To further confirm that this p38γ phosphorylation event is specifically due to p38β inhibition rather than off-target effects on other kinases, we treated Hut78 cells with Ralimetinib (LY2228820), a known ATP- competitive inhibitor of p38β (IC_50_=3.2 nM) [6]. Similar to Nilotinib, Ralimetinib (LY2228820) induced the appearance of the dual-phosphorylated p38γ band, though to a lesser extent (Figure 2B). Likewise, we validated this weaker band as p-p38γ using p38γ knock down approach. Ralimetinib also inhibits p38α (IC_50_=5.8 nM), though Nilotinib shows approximately 25-fold selectivity for p38β over p38α, as demonstrated by our *in vitro* kinase assay (Figure 1C).

### Potential EMT signal signature of Nilotinib in healthy PBMCs by single-cell RNA analysis

Given that compensatory p38γ activation can drive epithelial–mesenchymal transition (EMT) [13,14], we next examined whether Nilotinib triggers EMT-related pathways in healthy peripheral blood mononuclear cells (PBMCs). Single-cell RNA-sequencing of PBMCs treated with Nilotinib revealed simultaneous activation of KRAS signaling, EMT, and xenobiotic metabolism pathways (Figure 3A). We observed that EMT induction was confined to Treg and γδ T cell subsets.

To determine whether this p38γ-mediated EMT response extends beyond our experimental model, we next analyzed public datasets to assess the association between p38γ expression and EMT in clinical CTCL samples with large cell transformation. Public dataset [GSE113113] [15] analyses also support a role for p38γ—but not other p38 isoforms—in promoting EMT in CTCL with large-cell transformation (Figure 3B and Supplementary Figure S1). Our results implicate Nilotinib-induced EMT as a potential mechanism driving its immunotoxic or off-target effects.

To mitigate this potential toxicity, we sought a synergistic partner for Nilotinib. We first determined the cytotoxic IC_50_ of Nilotinib in Hut78 cells (Supplementary Figure S2A). Next, we evaluated its combination treatments with CSH71 [16], a selective p38γ inhibitor that targets the lipid-binding site (LBS).

Combination index (CI) analysis revealed strong synergy in CTCL cells (CI=0.69–0.74, Supplementary Figure S2B), but antagonism in healthy PBMCs (Supplementary Figure S2C), suggesting that dual inhibition of p38β and p38γ preferentially targets malignant cells. In contrast, combining Nilotinib with the ATP-site p38γ inhibitor F7/PIK75 [11] yielded only additive effects, but showed synergy when F7 in a higher dosage (Supplementary Figure S2).

### Clinical trials for Nilotinib in CML patients

To contextualize our preclinical findings, we reviewed the clinical trial landscape of Nilotinib in chronic myeloid leukemia (CML). A comprehensive overview of Phase I–IV studies (Table 2) demonstrates that Nilotinib has been extensively evaluated for efficacy and safety in both adult and pediatric CML populations [17–26]. Across multiple Phase II and III trials, Nilotinib consistently achieved higher rates of major molecular response (MMR) compared to Imatinib, particularly in newly diagnosed chronic-phase CML patients. In treatment-resistant or -intolerant cohorts, Nilotinib also produced meaningful cytogenetic and molecular responses. Pediatric trials revealed age-dependent pharmacokinetics and efficacy, while long-term follow-up studies confirmed durable responses with manageable toxicity [20–27]. Phase IV studies further confirmed long-term benefits, including sustained deep molecular responses (MR4.0 and MR4.5) and treatment-free remission in selected patients. Together, these data support Nilotinib as a potent second-generation tyrosine kinase inhibitor with an overall favorable benefit-risk profile in CML. Nevertheless, SAE rates ranged widely—from 3% to 60%—with higher toxicity observed in studies involving advanced disease. Notably, SAE rates are even higher in non-CML cancers such as gastrointestinal stromal tumors (GIST, Table 3), where Nilotinib acts as a multi-kinase inhibitor in the absence of BCR-ABL, increasing the risk of off-target toxicities driven by unintended pathway activation.

Given that p38β is upregulated in CML, its inhibition by Nilotinib may confer additional therapeutic benefit. However, clinical data in Table 4 (NCT02272777) [28,29] indicate a higher incidence of serious adverse events (SAEs) in Nilotinib-treated patients compared to those receiving Imatinib. This elevated toxicity likely reflects the broader impact of Nilotinib on dysregulated signaling networks involving both p38β and p38γ. Our findings show that Nilotinib-induced activation of p38γ occurs through inhibition of p38β (Figure 2A and 2B), offering a mechanistic explanation for adverse signaling and reduced therapeutic selectivity.

### ROR assessment and SAEs by Nilotinib in long-term clinical trials

To further assess safety, we analyzed 2024 (Q1–Q4) FDA Adverse Event Reporting System (FAERS) data [30], for three BCR-ABL inhibitors—Nilotinib, Imatinib, and Dasatinib. We used the Reporting Odds Ratio (ROR), a pharmacovigilance method recommended by the NIH [31–33], to detect adverse events (AEs) that occur more frequently with specific drugs. Our integrated analysis of clinical trial data highlights a consistent safety concern with Nilotinib use across cancer types. In Ph^+^ CML patients, the rate of SAEs exceeded 25% in multiple clinical trials, underscoring the need to weigh therapeutic efficacy against toxicity (Table 2). Notably, in Nilotinib-repurposed GIST trials, the incidence of SAEs was even higher than in CML trials (Table 3, Figure 4A) [clinicaltrials.gov].

Nilotinib showed greater toxicity in Ph^+^ CML patients compared to the other two TKIs (Figure 4B). Venn diagram analysis indicated that Imatinib was associated with the broadest range of adverse events (314 total signals), while Nilotinib had fewer (39 unique) but more severe signals. Dasatinib exhibited the fewest (17 unique), with nine AEs shared across all three drugs, suggesting potential class-related toxicities (Figure 4C). Applying a threshold of ROR>2 with at least three reports, we found that Nilotinib consistently showed higher ROR values than Imatinib and Dasatinib—especially for gastrointestinal, neurological, and cardiac events (Figure 4D). While Imatinib is linked to a broader range of AEs, Nilotinib appears to carry a greater risk of severe toxicities within the BCR-ABL inhibitor class.

### Relocation of p38β and p38γ genes following the t (9;22) (q34; Q11) chromosomal translocation

To better understand the regulation of p38β and p38γ in hematologic malignancies, we examined their genomic architecture and expression patterns. Notably, both MAPK11 (p38β) and MAPK12 (p38γ) are originally located on chromosome 22q11 [34]. In Philadelphia chromosome–positive (Ph^+^) CML, the t(9;22)(q34;q11) translocation relocates these genes to chromosome 9 [35,36], as illustrated in Figure 5A, placing them (p38s) near dysregulated epigenetic regulators such as EP300, but away from EHMT1. This genomic repositioning may contribute to aberrant signaling and altered chromatin regulation in leukemic cells.

To determine whether this structural rearrangement translates into functional changes, we analyzed public RNA-Seq dataset of leukemia samples [37]. The results revealed elevated expression of p38β and p38γ in leukemia samples, supporting their potential roles in leukemogenesis and in mediating the cellular response to Nilotinib relative to non-cancerous samples (Figure 5B). The translocation also shifts epigenetic regulators such as EHMT1, which is upregulated together with its isoform EHMT2 in Ph^+^ CML (Figure 5C). In addition, EP300 expression level has no significant changes (Supplementary Figure S4).

### Clinical trial of combination of Nilotinib and Paclitaxel

Paclitaxel selectively inhibits angiogenesis at ultra-low picomolar concentrations without disrupting microtubule assembly, supporting its clinical potential as a continuous low-dose anti-cancer strategy [38]. Table 6 indicated this promising combination (Nilotinib + Paclitaxel) in a published Phase I clinical trial (NCT02379416) [39], which demonstrated both safety and preliminary efficacy, including tumor regression in patients resistant to prior taxane (e.g., Paclitaxel) therapy. Notably, the trial also reported a reduced incidence of peripheral neuropathy—a common and debilitating side effect of Paclitaxel—suggesting a potential neuroprotective benefit of Nilotinib [40]. Upon these encouraging results, a Phase II trial (NCT05554341), known as ComboMATCH (EAY191-E4) [41] and sponsored by the National Cancer Institute (NCI) through the Eastern Cooperative Oncology Group - American College of Radiology Imaging Network (ECOG-ACRIN) Cancer Research Group, is currently underway to further assess the combination’s therapeutic potential in advanced solid tumors. However, clinical trials evaluating the Nilotinib and Paclitaxel combination in hematologic malignancies have not yet been initiated—a gap our research aims to address.

Preclinical studies using human tumor cell lines have shown that combining Nilotinib with Paclitaxel, a taxane-based chemotherapeutic agent, produces synergistic cytotoxic effects, killing cancer cells more effectively than either drug alone [39,40,42–54]. We also demonstrated that the IC_50_ of Paclitaxel in CTCL Hut78 cells is 2nM (Supplementary Figure S5), however with a higher dosage of Paclitaxel, Hut78 cells showed increase of cell proliferation. Consistent with this, our cytotoxicity assays demonstrated synergistic effects of Nilotinib + Paclitaxel in acute myeloid leukemia (AML) Molm13 cells (Supplementary Figure S6).

Moreover, we found that Paclitaxel directly binds to p38γ, as validated by mass spectrometry (Figure 6), suggesting that it may act as a p38γ inhibitor. Depending on experimental conditions, one to three Paclitaxel molecules were detected bound to each p38γ protein. By functioning as a p38γ inhibitor, Paclitaxel may counteract the elevated p38γ activity induced by Nilotinib’s p38β inhibition.

## Discussion

### Nilotinib-induced activation of p38γ through p38β inhibition

The higher rate of SAEs observed with Nilotinib in non-CML malignancies compared to CML is rooted in differences in disease biology. In CML, the characteristic t(9;22)(q34;q11) translocation produces the BCR-ABL fusion kinase—a direct driver of leukemogenesis and an ideal drug target. The development of Imatinib (Gleevec), a selective BCR-ABL inhibitor, revolutionized CML treatment and became a landmark in pharmacology, establishing the paradigm of targeted cancer therapy. Nilotinib, a second-generation BCR-ABL inhibitor, builds upon this success. However, Nilotinib also inhibits other kinases, including p38β. In cancers lacking the BCR-ABL fusion, such as GIST, Nilotinib’s multi-kinase activity may engage non-malignant signaling proteins, reducing efficacy and increasing off-target toxicities. Importantly, parallel elevation of p38β and p38γ expression is observed in cutaneous T-cell lymphoma (CTCL) [11] as well as in leukemia (Figure 5B). Our findings show that Nilotinib modulates oncogenic pathways in CTCL by targeting p38β and indirectly promoting p38γ activation—independent of the t(9;22) (q34;q11) translocation—highlighting a novel mechanistic axis that contributes to Nilotinib-associated toxicities across diverse cell types through a general p38 isoform compensation mechanism.

This mechanism appears broadly applicable, as supported by public literature. Similar higher molecular weight bands—indicative of p38γ activation—have been observed in Western blots of myoblasts (non- hematologic cells) treated with either Nilotinib or the p38β inhibitor SB203580 [12]. These studies also reported that Nilotinib promotes myoblast proliferation, thereby preventing the formation of mature myotubes. Notably, p38γ is highly expressed in skeletal muscle cells, of which myoblasts serve as their precursors. Although the functional significance of these bands was not explored [12], these findings support our conclusion that in cells with high p38γ expression, p38β inhibition triggers compensatory activation and phosphorylation of p38γ. This mechanism likely contributes to both cancer progression and Nilotinib-associated SAEs. Notably, two long-term ENESTnd studies (**E**valuating **N**ilotinib **E**fficacy and **S**afety in **n**ewly **d**iagnosed Ph^+^ Chronic Myeloid Leukemia in chronic phase, >10 years follow-up) reported significantly higher rates of cardiovascular events (CVEs) and hepatotoxicity in Nilotinib-treated patients compared to those receiving Imatinib [55,56].

We further demonstrate that p38γ inhibitors synergize with Nilotinib to enhance anti-tumor activity across multiple cancer types [6], including AML (Supplementary Figure S6), suggesting a potential strategy to improve both the safety and efficacy of Nilotinib in CML and solid tumors. Clinical studies also support the promise of this approach. A Phase I trial (NCT02379416) reported favorable safety and preliminary efficacy of the Nilotinib + Paclitaxel combination, including reduced incidence of Paclitaxel-induced peripheral neuropathy. A follow-up Phase II study (NCT05554341, ComboMATCH) is currently evaluating this combination in advanced solid tumors. However, its clinical efficacy in hematologic malignancies remains unexplored, emphasizing the translational importance of our findings.

To assess whether Nilotinib is disproportionately associated with specific toxicities, we performed a pharmacovigilance analysis using the ROR, a standard metric that quantifies the likelihood of an adverse event being reported for a particular drug compared to others. A significantly elevated ROR flags a potential safety concern that warrants further investigation.

### EMT

EMT is a transformative biological process where epithelial cells lose their apical-basal polarity and loosen cell–cell connections, adopting the shape and invasive traits of mesenchymal cells to migrate through the extracellular matrix [57]. KRAS activation can trigger EMT via pathways like MAPK/ERK and PI3K/AKT, influencing transcription factors such as SNAIL [13], SLUG, and TWIST to facilitate this transition. In our single-cell analysis (Figure 3A), we observed that only two T cell populations—Tregs and γδ T cells—are prone to exhibit EMT signatures among Nilotinib-treated PBMC population. While in general, Treg cells do not undergo full EMT, they can adopt EMT-like transcriptional programs to enable tissue infiltration and motility, largely via TGF-β/SMAD signaling. γδ T cells are particularly prone to EMT-like reprogramming, especially within tumor microenvironments rich in TGF-β, enabling tissue infiltration, matrix remodeling, and—depending on the context—either tumor progression or immune evasion [58–62]. In this context, Nilotinib-induced EMT in PBMCs is likely driven by its inhibition of p38β, which in turn leads to compensatory activation of p38γ. Notably, p38γ MAPK has been shown to promote EMT and the expansion of cancer stem cell (CSC) populations in breast cancer cells [14], supporting the relevance of this mechanism across cell types. EMT is associated with harmful outcomes in adult tissues, including its involvement in pathological processes such as fibrosis and cancer metastasis.

### Chromosomal translocation and pathway dysregulation

Chromosomal rearrangements can profoundly alter gene proximity and regulatory landscapes, contributing to oncogenesis. In Ph^+^ CML, the characteristic BCR-ABL translocation (t(9;22)(q34;q11)) not only produces the BCR-ABL fusion kinase but also repositions the p38β and p38γ genes from chromosome 22 near genes on chromosome 9, including CHEK2, CBX6, and EP300. The BCR-ABL fusion locus is likewise positioned near additional leukemia-associated genes such as LAMC3, NOTCH1, and EHMT1 (Figure 5A). This complex rearrangement may foster an altered crosstalk between kinase signaling and epigenetic regulation. Although the precise mechanism by which p38β inhibition triggers compensatory p38γ phosphorylation remains under investigation, the chromosomal context suggests that these interactions are embedded within broader dysregulated networks. Our findings underscore the complexity of chromosomal rearrangements in cancer and highlight the importance of understanding their broader impact beyond fusion gene generation, particularly as it relates to therapeutic resistance and disease progression.

Among the affected epigenetic regulators, histone acetyltransferase EP300 [63] and euchromatic histone-lysine N-methyltransferase 1 (EHMT1) are also repositioned by the translocation (Figure 5A). Expression profiling reveals that while EP300—a well-established tumor suppressor [64]—is not significantly downregulated (Supplementary Figure S4), EHMT1, which promotes leukemic stemness [65,66], is markedly upregulated in Ph^+^ CML patients (Figure 5C). EHMT2 (G9a, located on chromosome 6), is similarly upregulated. EHMT1, functioning as a heterodimer with EHMT2, catalyzes H3K9 mono- and dimethylation, generating repressive chromatin marks that silence tumor suppressor genes and sustain leukemic stemness [67,68]. Our data confirmed elevated EHMT1 expression (p<0.001), further implicating epigenetic imbalance in CML pathogenesis. We hypothesize that the t(9;22)(q34;q11) translocation not only drives oncogenesis through BCR-ABL fusion kinase activity but also reshapes the genomic and epigenetic landscape of Ph^+^ leukemia. The repositioning and dysregulation of key signaling and epigenetic regulators, including p38γ/p38β, EHMT1, and others—create a permissive environment for aberrant pathway crosstalk and leukemic stemness.

### Clinical implications

Our data further reveal that Paclitaxel directly binds to p38γ and may functionally act as a p38γ inhibitor, counteracting the Nilotinib-induced activation of p38γ and contributing to the observed synergy in CTCL and AML models. Importantly, our study uncovers a previously unrecognized mechanism: Nilotinib potently inhibits p38β, which in turn drives compensatory hyperactivation of p38γ. This unintended activation of p38γ may underlie many of the EMT- related phenotypes and serious adverse effects observed in Nilotinib-treated patients, especially in cancers lacking BCR-ABL. These findings offer a compelling mechanistic rationale for applying Nilotinib in combination with p38γ inhibitors to neutralize its p38γ-driven toxicities and enhance therapeutic efficacy across both hematologic malignancies and solid tumors. Although the Nilotinib + Paclitaxel combination exhibits strong synergistic anti-cancer activity, known risks of neurotoxicity and cardiotoxicity for both agents warrant caution. Our results suggest that combining Nilotinib with safer p38γ-selective inhibitors could offer a more favorable therapeutic window. In this regard, FDA-approved pirfenidone—a known p38γ inhibitor [69,70] —demonstrates synergy with Nilotinib in CTCL (Figure 7) and may help mitigate its adverse effects. Additionally, more selective alternatives such as the LBS-targeting p38γ inhibitor CSH71 [16], Pirfenidone [71], or the ATP-site inhibitor F7/PIK75 [11] represent promising strategies to optimize clinical outcomes while minimizing toxicity.

## Materials and Methods

### High-throughput screening for p38β inhibitor from FDA-approved drug library

We used an *in vitro* kinase assay to screen a library of 1,443 FDA-approved compounds (1,393 in DMSO, 50 in water) to identify p38β inhibitors. The library, in 96-well format, was purchased from Selleck Chemicals (Houston, TX, USA).

### P38 kinase assays

*In vitro* kinase assays were performed using the ADP-Glo kit (Promega, WI) (Promega) as previously described [22]. Briefly, human recombinant p38 α, β or δ protein (active full-length) expressed by baculovirus in sf9 insect cells (Signal Chem, Vancouver, Canada) was homogenized in 96-well white opaque microplates with kinase buffer (40 mM Tris-HCl (pH 7.5), 20 mM MgCl2, 0.1 mg/mL BSA, and 50 μM DTT). p38 kinase was preincubated with compounds as indicated in a dose-dependent manner for 10 min. Next, synthetic peptide substrates (IPTTPITTTYFFFKKK) were added to the mixture at a final concentration of 0.2 μg/μL followed by ATP at various concentrations.

### Viability assays using trypan blue exclusion and CellTiterGlo Cell Viability Assay

Cell viability was calculated by diluting cell suspensions 1:1 in 0.4% trypan blue solution (Sigma-Aldrich, St. Louis, MO) and counting the number of viable cells using a TC20 automated cell counter (Bio-Rad, Irvine, CA) that automatically excludes non-viable cells. CellTiterGlo Cell Viability Assay (Promega) was used as previously described [22]. All data points represent the average of three experiments. SS patients’ cells acquisition was approved by UMAS (IRB#204503, MTA#25468).

### Single cell RNA sequencing analysis

Cell number and viability were measured using a TC20 Automated Cell Counter (Bio-Rad), and only samples with ≥80% viability were processed. Both CSH71- or Nilotinib-treated cells were loaded onto a Chromium Controller (10x Genomics), targeting 2,000–5,000 cells per lane. Single-cell 3′ RNA-seq libraries were prepared using the Chromium Single Cell 3′ v2 Reagent Kit (10x Genomics) following the manufacturer’s protocol and sequenced on a HiSeq 2500 system (Illumina) with a depth of 50,000–100,000 reads per cell. Raw sequencing data were processed using the Cell Ranger pipeline (v2.0, 10x Genomics) and aligned to the mm10 reference genome. Data analysis was performed with the Seurat package in R. Low-quality cells (greater than 10–20% mitochondrial reads and less than 200 detectable genes) were excluded. Data were normalized, scaled, and analyzed using principal component analysis of highly variable genes, followed by clustering and visualization with t-SNE or UMAP. Cluster-specific marker genes were identified to generate heatmaps and feature plots. Gene set enrichment analysis (GSEA) was performed using Hallmark gene sets from MSigDB.

### Western blot analysis and evaluation of p38β and p38γ gene silencing by shRNA expression

Hut 78 cell culture and gene silencing by shRNA were performed as previously described [23]. MISSION^®^ shRNA Lentiviral Transduction Particles (company validated) shRNAs in pLKO.1- puro shRNA vector that targets the human p38β (MAPK11), p38γ gene (MAPK12) and a scramble Transduction Particles (pLKO.1-puro shRNA vector only) were purchased from Sigma. All antibodies were procured from Cell Signaling, Danvers, MA, including p38α (Cat# 9218), p38β (2339), p38γ (2307), p38δ (2308), p-p38 T180/Y182 (4511), and GAPDH (2118).

### Analysis of Paclitaxel binding to p38γ by native mass spectrometry

Recombinant p38γ (450 μM) was buffer-exchanged into 50 mM ammonium acetate (pH 7.5) using an Amicon Ultra 10 kDa cutoff filter and diluted to a final concentration of ~9 μM. Potential ligands were prepared at 10 mM in HPLC/MS-grade water (Vinorelbine ditartrate, Betaxolol HCl, DL-Carnitine HCl, Sildenafil citrate, myo-Inositol, Oclacitinib maleate) or ethanol (remaining ligands). Ligands (1 μL) were added to 50 μL aliquots of p38γ to achieve final concentrations of 9 μM p38γ and 200 μM ligand.

Samples were analyzed on an Agilent 6520 QTOF mass spectrometer operated in high mass mode, with flow injection at 100 μL/min using 50 mM ammonium acetate (pH 7.5) as carrier solvent. Instrument parameters were optimized for native mass spectrometry. Spectra were averaged, and background spectra (collected during protein-free elution) were subtracted. Data in the m/z 2500–4500 range were deconvoluted using UniDec.

### Statistical analysis

All experimental data are presented as mean ± standard error of the mean (SEM), unless otherwise indicated. Statistical significance of differences, such as in cell viability assays and mRNA expression of target genes, was assessed using the Student’s t-test (SPSS; IBM, Armonk, NY) or one-way analysis of variance (ANOVA) (GraphPad PRISM, version 3.0, GraphPad). Differences were considered significant at P < 0.05.

## Supplementary Material

Suppl. materials

## Figures and Tables

**Figure 1.**
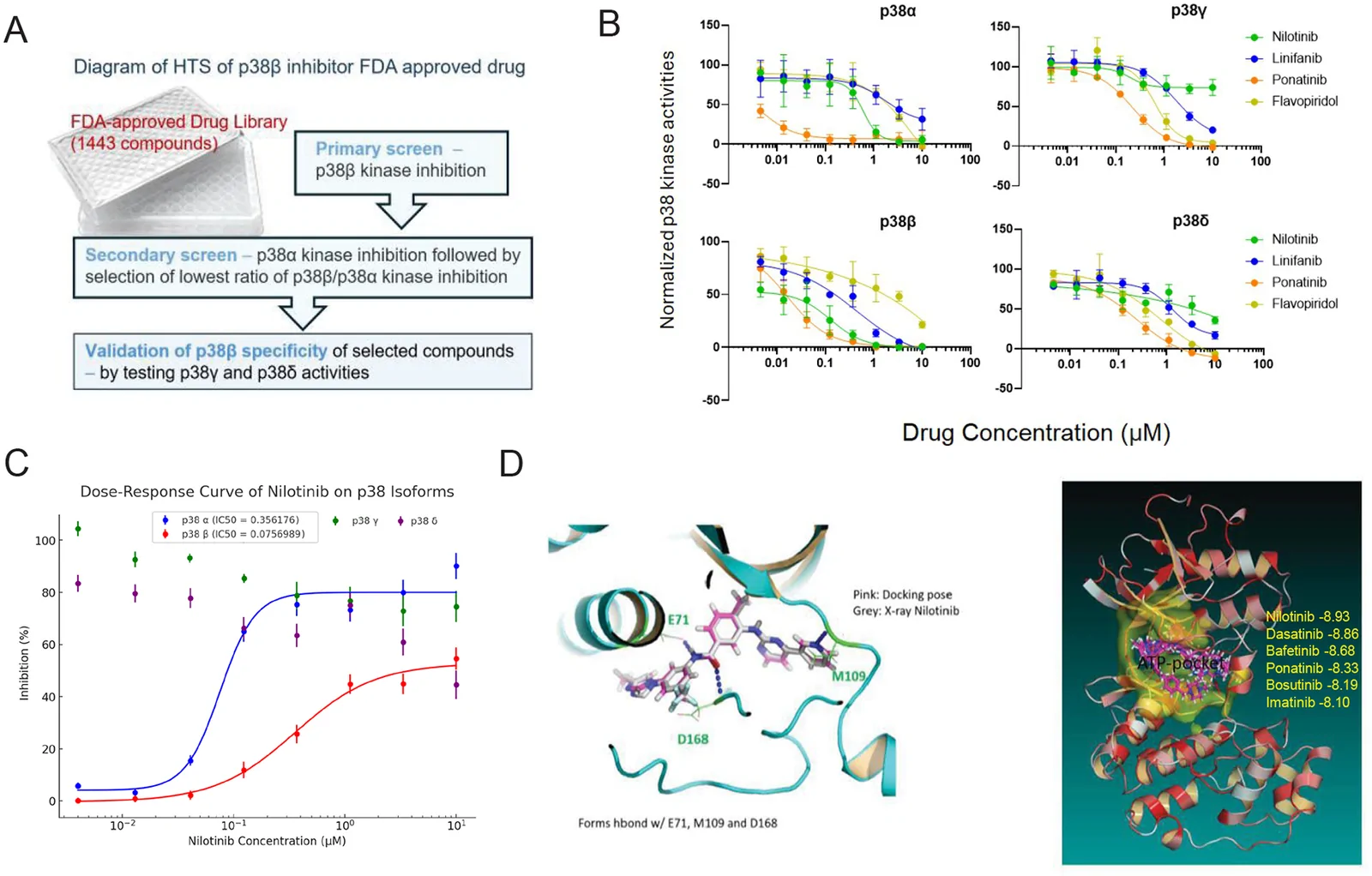
Nilotinib is selected as p38β inhibitor. (**A**)Diagram of High throughput screening (HTS) of p38β inhibitor from an FDA approved drug library (Selleck). (**B**) Effects of 4 FDA approved drugs (Flavopiridol, Linifanib, Ponatinib and Nilotinib) on kinase activities of 4 p38 isoforms. Nilotinib, a Bcr-Abl inhibitor, stood out as a potent p38β specific inhibitor, with IC_50_=14nM (red color) compared to other p38 isoforms. Inhibitory IC_50_ (μM) of Nilotinib for other 3 isoforms of p38 kinase activity listed in the Table 1. Nilotinib, a Bcr-Abl inhibitor, a potent p38β specific inhibitor in comparison of 3 other selected compounds. (**C**) Kinase activity assays of p38 MAPK isoforms. Nilotinib selectively inhibits p38β kinase activity (IC_50_=0.014–0.075 μM) with approximately 25-fold greater potency than p38α (IC_50_=0.356 μM), while showing minimal inhibition of p38γ and p38δ. These results demonstrate that Nilotinib is a more specific inhibitor of p38β compared to p38α. (**D**) Docking pose on p38β of Tyrosine Kinase inhibitors (TKI). Left: Docking pose of Nilotinib with p38β superimposed on X-ray crystallography data. Right: Nilotinib −8.93 showed the best docking score on p38β (−8.93 kcal/mol) in comparison of the other 5 TKIs: Dasatinib, Bafetinib, Ponatinib, Bosutinib and Imatinib, all of which binds p38β in the ATP-binding pocket. Imatinib is −8.10kcal/mol.

**Figure 2.**
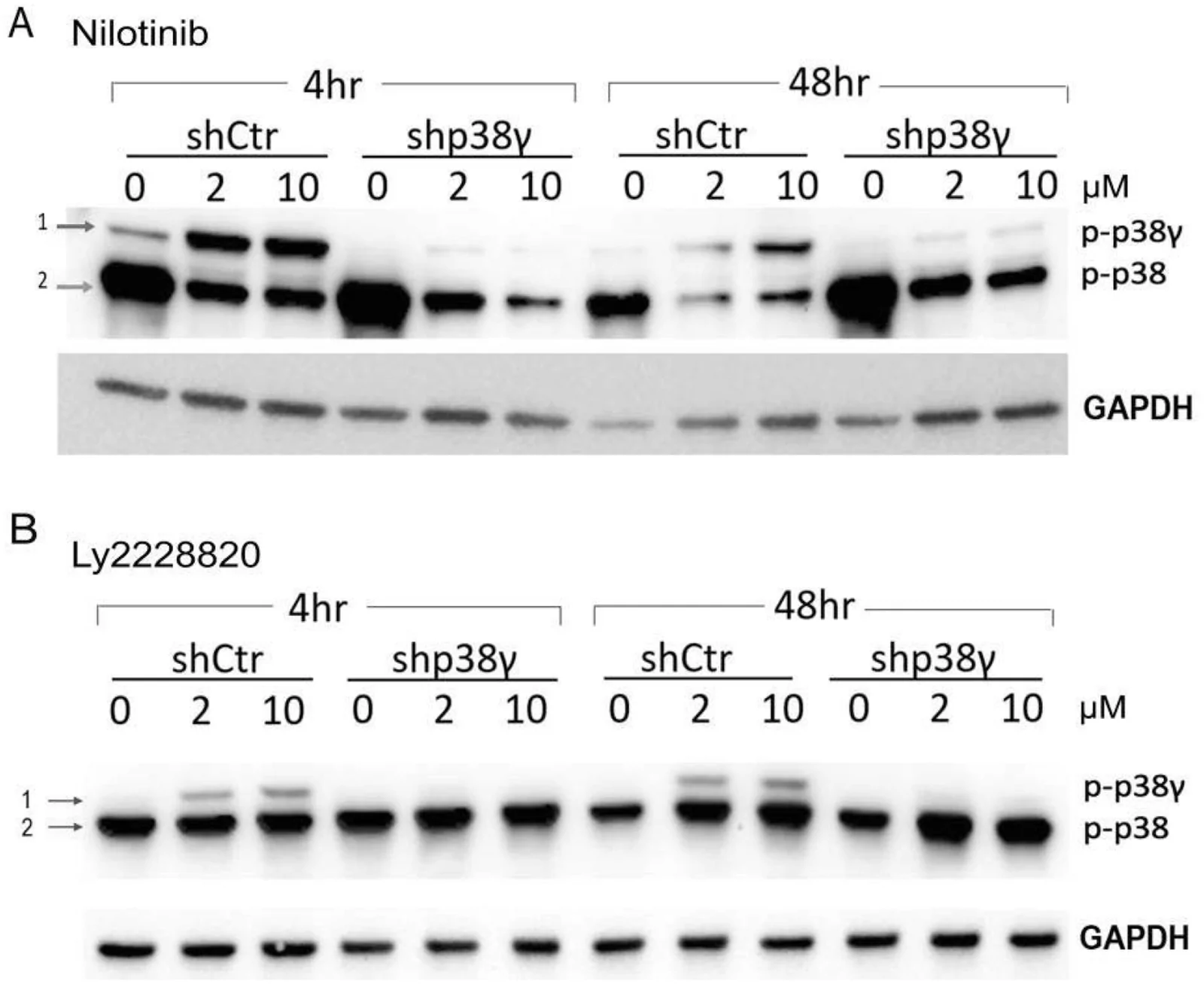
Nilotinib induces dual phosphorylation of p38γ in CTCL cells. (**A**) Scramble control (shCtrl) and p38γ knockdown Hut78 cells were treated with Nilotinib for 4 hours and 48 hours. A higher molecular weight band (Band 1), corresponding to the dually phosphorylated form of p38γ (p-p38γ), was detected in shCtrl cells but disappeared in p38γ knockdown cells, confirming its identity. (**B**) A similar experiment was conducted using the p38β/α inhibitor LY2228820. Treatment induced a weaker but distinct Band 1 in shCtrl cells, which was also abolished in p38γ knockdown cells. Note: p38γ has a higher molecular weight (367 amino acids) compared to other p38 isoforms (~360 amino acids), allowing clear resolution of p-p38γ (Band 1) from other isoforms (Band 2).

**Figure 3.**
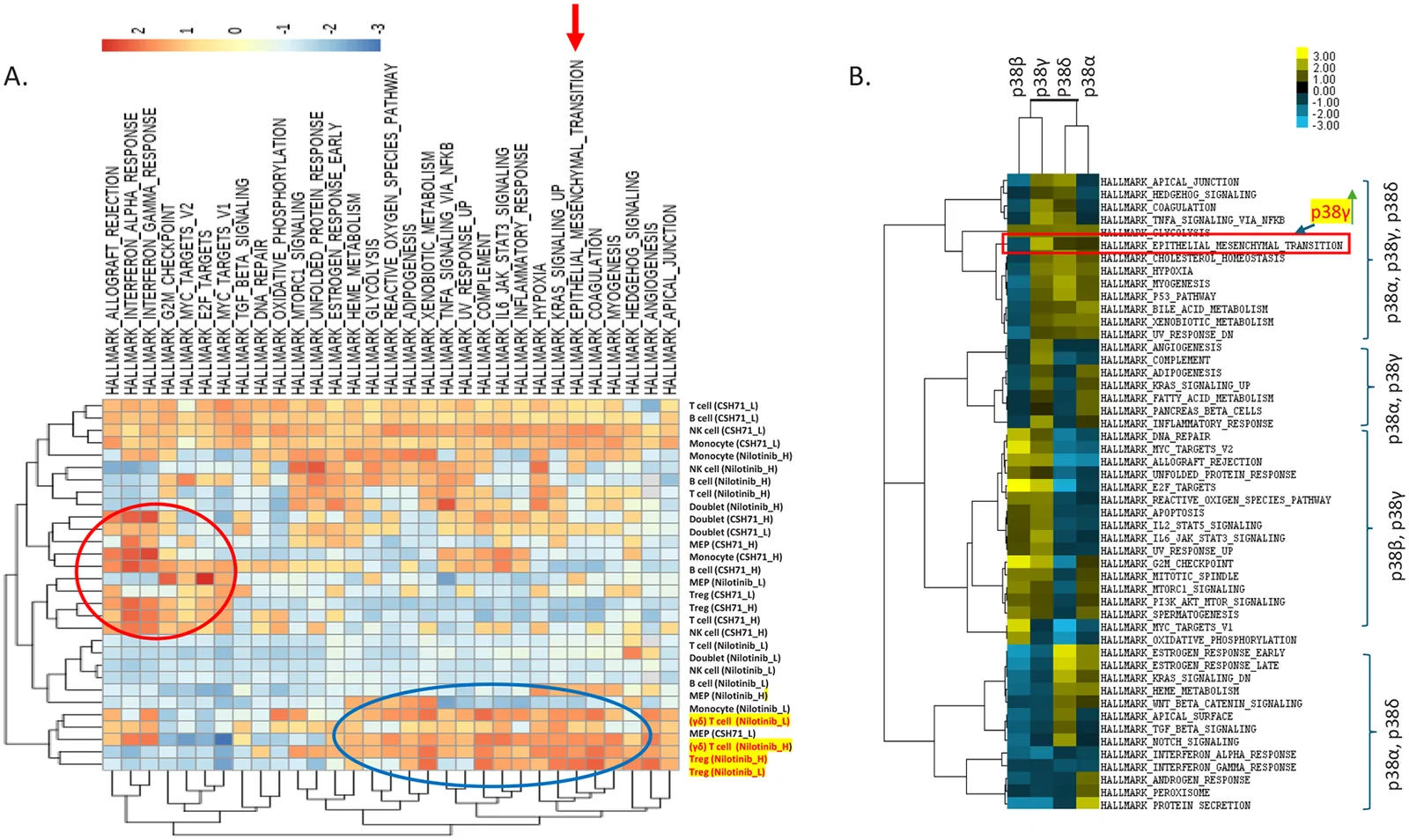
Single-cell RNA sequencing reveals distinct immune cell responses to Nilotinib and CSH71 treatments. (**A**) Single-cell transcriptomic analysis of PBMCs treated with Nilotinib or CSH71 at low (L) and high (H) concentrations identifies major immune cell populations, including T cells, B cells, NK cells, monocytes, Tregs, γδ T cells, and megakaryocyte-erythroid progenitors (MEPs). Treatment-specific clustering highlights transcriptional shifts, including enrichment of EMT-like signatures in Tregs and γδ T cells upon Nilotinib treatment and interferon response pathways with CSH71. Doublet populations and cell-type-specific responses are annotated by treatment condition. (**B**) A Public dataset analyses [GSE113113, https://www.ncbi.nlm.nih.gov/geo/] indicated that it is p38γ—but not other p38 isoforms—promoting EMT in CTCL with large cell transformation (LCT).

**Figure 4.**
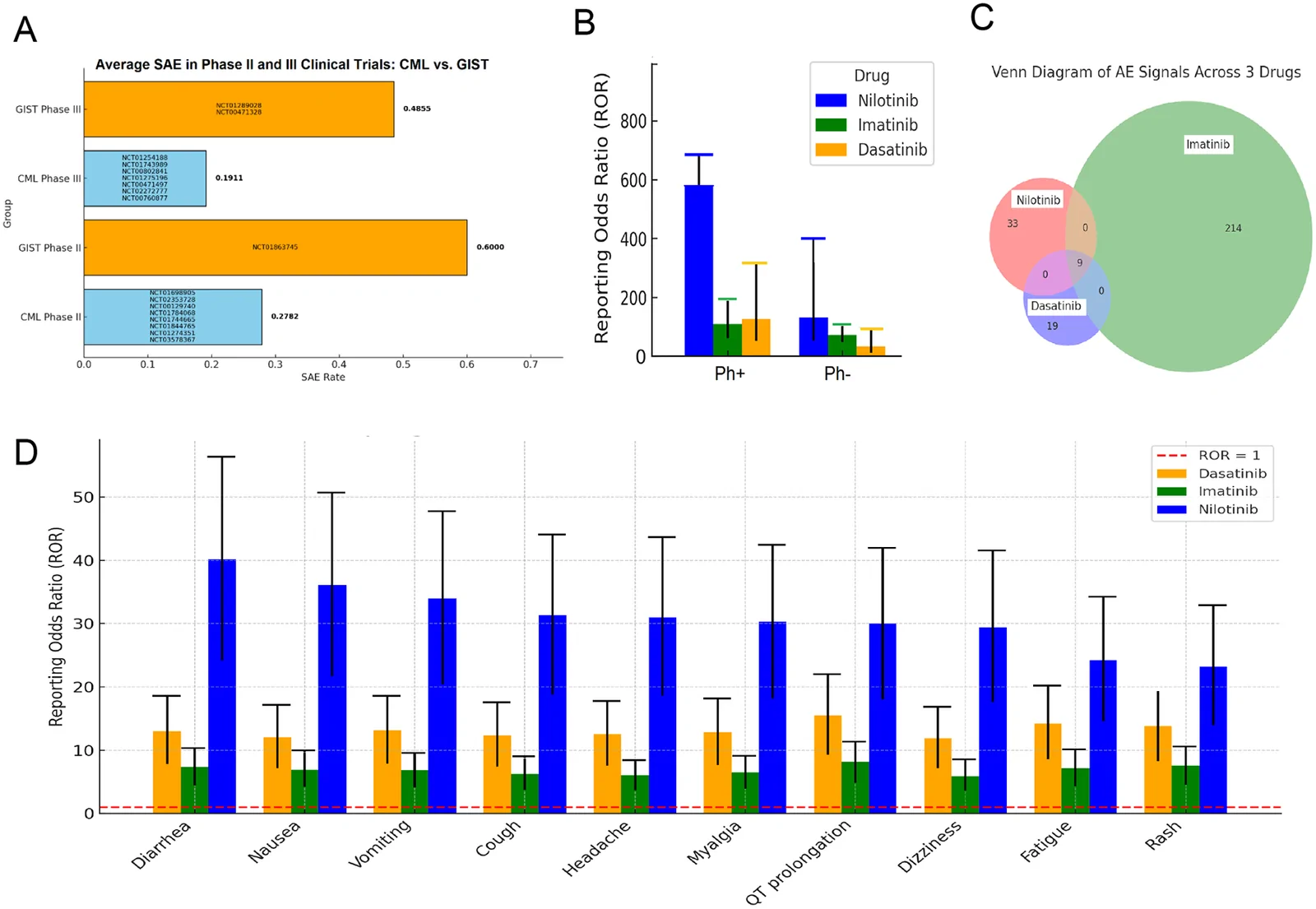
Comparative safety analysis of BCR-ABL inhibitors using clinical trial data and FAERS pharmacovigilance signals. (**A**) Incidence of serious adverse events (SAEs) across clinical trials for Nilotinib in CML and non-CML malignancies, showing a higher SAE rate in non-CML (e.g., GIST) settings. For example, a CML trial with 200 patients and 38 SAEs yields an SAE rate of 0.19, whereas a GIST trial with 150 patients and 62 SAEs yields a rate of 0.413. (**B**) Total number of adverse event (AE) signals associated with Nilotinib, Imatinib, and Dasatinib, based on 2024 Q1–Q4 FAERS data. The analysis shows that Imatinib is linked to the broadest range of AEs, while Nilotinib is associated with fewer but more severe events. (**C**) Venn diagram illustrating the overlap and uniqueness of reported AEs for Nilotinib, Imatinib, and Dasatinib. Nilotinib and Dasatinib share some common AEs with Imatinib but also exhibit distinct AE profiles, underscoring potential differences in off-target effects. (**D**) Reporting Odds Ratio (ROR) analysis comparing Nilotinib to Imatinib and Dasatinib. Nilotinib shows significantly elevated ROR values, particularly for gastrointestinal, neurological, and cardiac toxicities, indicating a higher relative risk of severe adverse events. Note: Reporting Odds Ratio (ROR) is a pharmacovigilance metric used to detect whether a particular adverse event (AE) is reported more frequently for a specific drug compared to others in a large database (like FAERS). Data indicates 10 AEs are reported much more frequently for Nilotinib than for Imatinib and Dasatinib.

**Figure 5.**
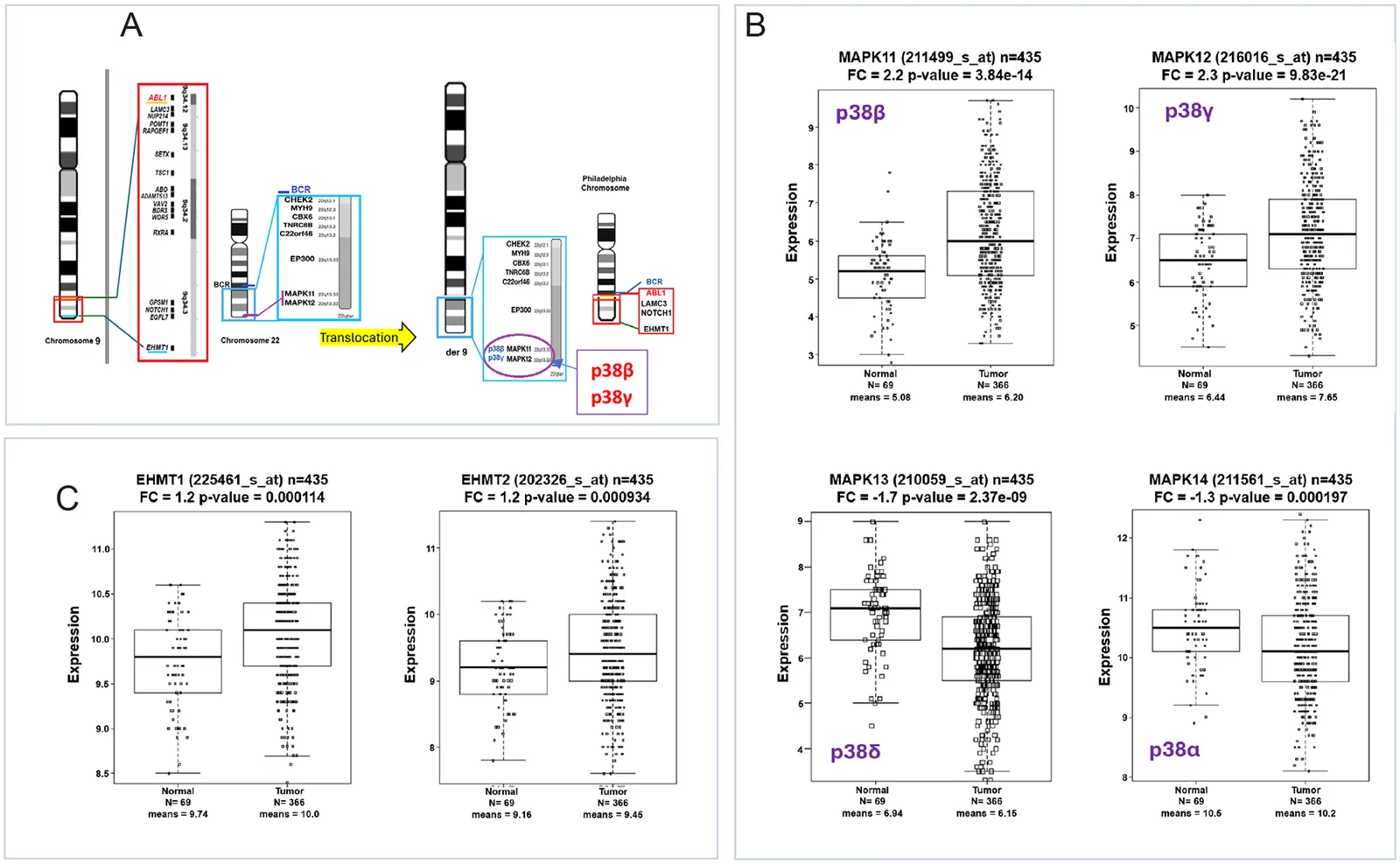
Chromosomal translocation t(9;22)(q34;q11) alters genomic positioning and expression of p38 isoforms and epigenetic regulators in Ph^+^ CML. (**A**)Schematic representation of the t(9;22)(q34;q11) translocation in chronic myeloid leukemia (CML), illustrating the repositioning of genes including MAPK11 (p38β), MAPK12 (p38γ), EP300, and EHMT1. The rearrangement relocates these genes from chromosome 22 to chromosome 9, placing them near additional oncogenic loci such as CHEK2, CBX6, LAMC3, and NOTCH1, potentially altering transcriptional regulation and chromatin architecture. (**B**) Public database analysis for RNA-Seq in leukemia samples (Mills *et al*. 2009 [37], GSE15061) showing significantly elevated expression of p38β (MAPK11) and p38γ (MAPK12), and reduced expression of p38α (MAPK14) and p38δ (MAPK13) in leukemia samples compared to non- cancerous samples. These findings suggest that the chromosomal translocation may contribute to dysregulated MAPK signaling in Ph^+^ CML. (**C**) Expression profiling of epigenetic regulators shows marked upregulation of EHMT1 and EHMT2 (G9a) compared to non-cancerous samples. The elevated EHMT1/2 expression supports a repressive chromatin environment conducive to leukemic stemness and therapy resistance.

**Figure 6.**
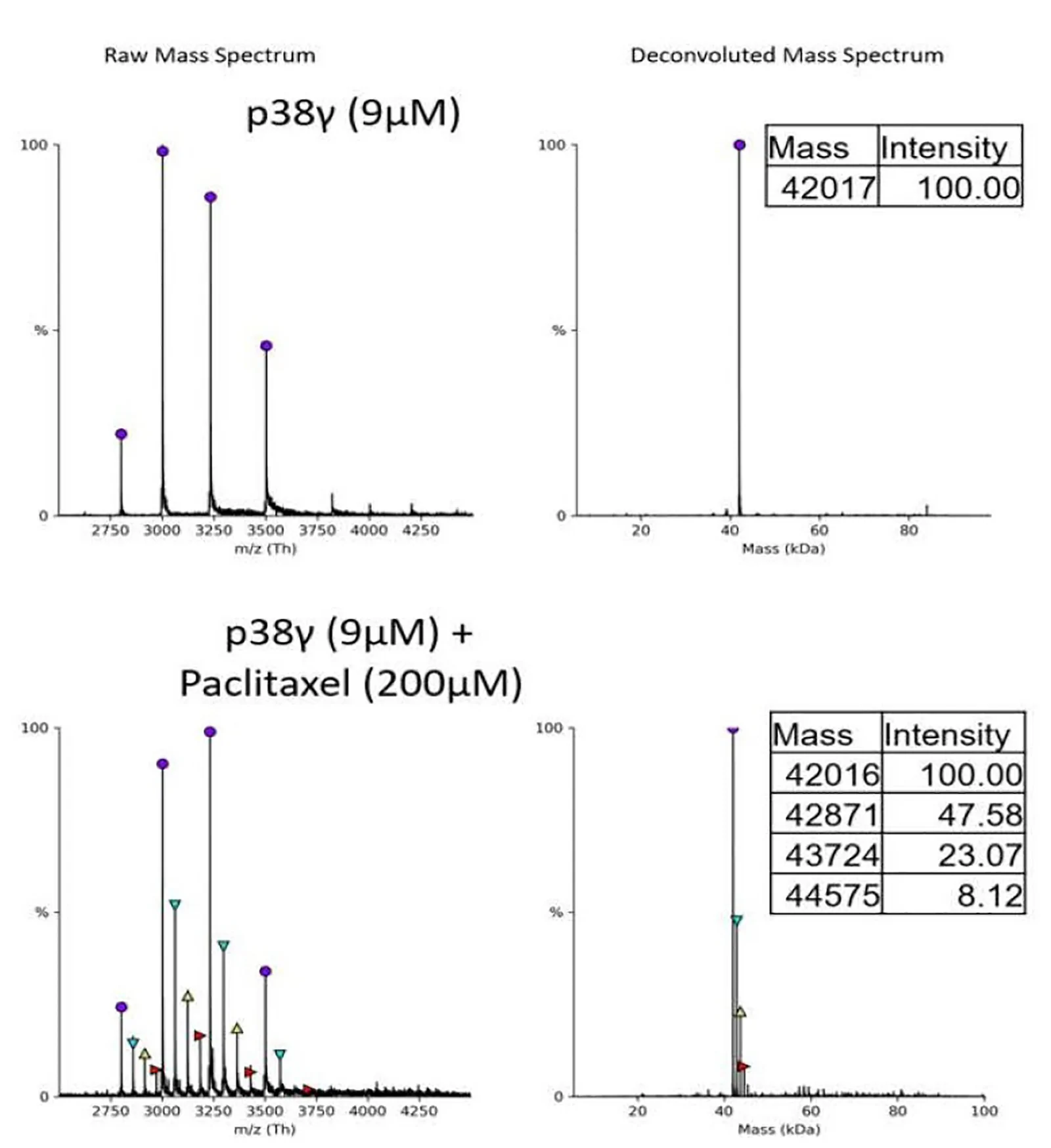
Native mass spectrometry confirms direct binding of Paclitaxel to p38γ. **Left panels** show raw mass spectra; **right panels** show corresponding deconvoluted spectra. **Top panel:** Deconvoluted native mass spectrum of recombinant p38γ (9 μM) alone displays the expected molecular mass of unbound protein. **Bottom panel:** Deconvoluted spectrum of p38γ incubated with Paclitaxel (200 μM) reveals a distinct mass shift consistent with the formation of a p38γ–Paclitaxel complex. Data validate Paclitaxel as a direct binder of p38γ and provide biophysical support for its potential role in counteracting Nilotinib-induced p38γ upregulation, as observed in synergy experiments.

**Figure 7.**
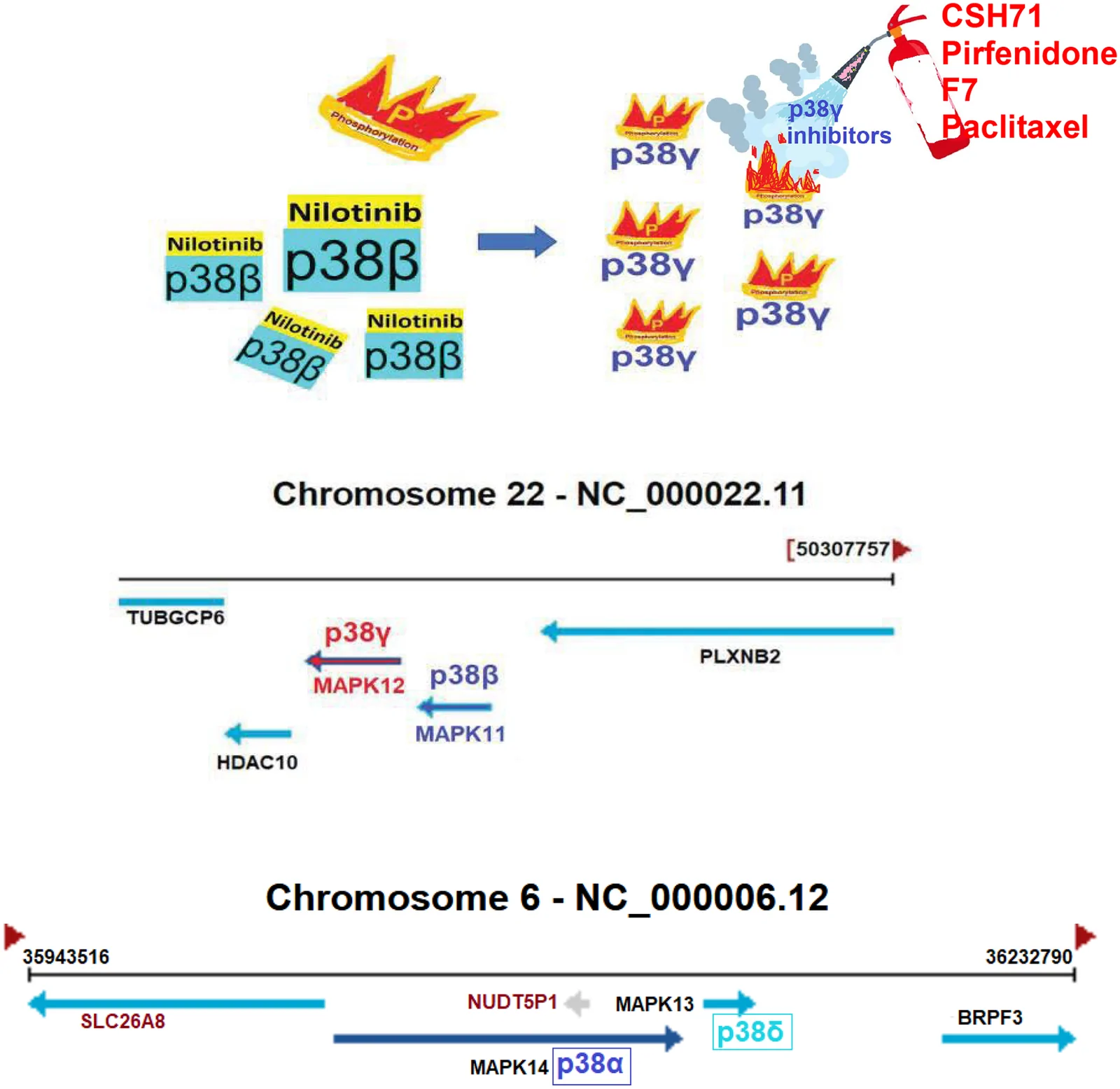
Illustration of compensatory activation of p38γ by Nilotinib and genomic localization of p38 MAPK isoforms. (**A**) Schematic depicting the compensatory activation of p38γ following p38β inhibition in cells expressed p38γ. Nilotinib binds to and inhibits p38β, leading to increased phosphorylation and activation of p38γ, a response that can be attenuated by p38γ-specific inhibitors to alleviate downstream stress signaling. (**B**) Genomic mapping shows that p38γ (MAPK12) and p38β (MAPK11) are located adjacent to each other on chromosome 22q13.33, suggesting potential transcriptional or regulatory linkage. (**C**) p38α (MAPK14) and p38δ (MAPK13) are clustered together on chromosome 6p21.31, indicating conserved genomic organization among p38 MAPK family members.

**Table 1.** IC_50_ (μM) of p38 kinase activities of 4 FDA approved drugs. Nilotinib, a BCR-ABL inhibitor, is also a potent p38β specific inhibitor which binds p38β in the ATP- binding pocket. Nilotinib specifically inhibits p38β with IC_50_=14nM (red) compared to other p38 isoforms. Inhibitory IC_50_ (μM) of Nilotinib for other 3 isoforms of p38 kinase activity are p38α=0.35, p38γ>10 and p38δ=4.45. Ponatinib is a pan p38 inhibitor but with a potent inhibition to p38α IC_50_=0.003.

	Flavopiridol	Linifanib	Nilotinib	Ponatinib
**P38α**	1.34	2.18	0.35	0.003
**P38β**	1.82	0.12	0.014	0.015
**P38γ**	0.65	2.54	>10	0.23
**P38δ**	0.45	1.21	4.45	0.18

**Table 2.** Summary of clinical trials evaluating Nilotinib in Chronic Myeloid Leukemia (CML). A summary of 21 clinical trials assessing the efficacy, safety, and pharmacokinetics of Nilotinib across various phases in CML patients. Key outcomes include molecular response rates, cytogenetic responses, and sustained remission data, along with the reported incidence of serious adverse events (SAEs), expressed as a proportion per trial. Trials include both adult and pediatric populations, with comparisons to Imatinib in several Phase III studies.

Clinical Trial	Phase	Aim	Result	Cancer Type	SAE
NCT00109707	I/II	Evaluate efficacy, safety, and PK of Nilotinib in CML/ALL patients across six groups	Major cytogenetic response after 7.5 years: 59.5% (CML-CP, prior Imatinib), 32.1% (CML-AP, prior Imatinib)	AML, CML	0.454
NCT01077544	I	Assess PK, safety, and activity of Nilotinib in pediatric Ph^+^ CML or Ph^+^ ALL patients	MMR achieved in 1/5 patients <10 years, and 2/6 patients aged 10–18	AML, CML	0.25
NCT01698905	II	Evaluate if Nilotinib can be safely discontinued in CML patients	57.9% achieved treatment-free remission within 48 weeks	CML	0.208
NCT02353728	II	Assess nilotinib’s effect on leukemic stem cells in newly diagnosed CML	—	CML	0.312
NCT00129740	II	Determine if Nilotinib controls chronic phase CML	131/148 achieved complete cytogenetic response; 23/148 achieved complete molecular response	CML	0.317
NCT01784068	II	Evaluate safety of Nilotinib cessation in CML	51.6% achieved MMR at 48 weeks (n=190)	CML	0.186
NCT01744665	II	Assess relapse-free survival post-Nilotinib in MR4.5 patients	Median relapse-free survival: 21.4 weeks	CML	0.322
NCT01844765	II	Evaluate long-term safety and PK of Nilotinib in pediatric Ph^+^ CML	MMR at 6 cycles in resistant pediatric CML: 39.4%; at 12 cycles in newly diagnosed: 64%	CML	0.259
NCT01274351	II	Study Nilotinib as first-line therapy in adult Ph^+^ CML-CP	66.1% achieved MMR at 12 months	CML	0.384
NCT03578367	II	Compare Nilotinib vs Asciminib ± Imatinib in pretreated CML-CP	MR4.5 at 48 weeks: Asciminib60+Imatinib400=19%, Asciminib40+Imatinib400=28.6%, Imatinib alone=0%, Nilotinib300=14.3%	CML	0.238
NCT01254188	III	Evaluate efficacy and safety of Nilotinib in newly diagnosed CML	70.8% achieved MMR at 12 months	CML	0.230
NCT01743989	III	Assess optimal Nilotinib treatment duration in Ph^+^ CML	Sustained remission: 31.9% (24 months), 37.5% (36 months)	CML	0.220
NCT00802841	III	Compare efficacy of Nilotinib vs Imatinib in CML	Complete cytogenetic response: 50% (Nilotinib) vs. 42.1% (Imatinib)	CML	0.115
NCT01275196	III	Compare Nilotinib vs Imatinib in Chinese CML-CP patients	MMR: 52.2% (Nilotinib) vs. 27.8% (Imatinib)	CML	0.083
NCT00471497	III	Compare two Nilotinib doses vs Imatinib in newly diagnosed Ph^+^ CML-CP	MMR at 12 months: 44.3% (Nilotinib 300 mg), 42.7% (400 mg), 22.3% (Imatinib)	CML	0.455
NCT02272777	III	Extended comparison of Nilotinib vs Imatinib in Chinese CML-CP patients	AEs: 84/113 (Nilotinib) vs. 82/112 (Imatinib); SAEs: 3/113 vs. 1/112	CML	0.027
NCT00760877	III	Compare cumulative CMR between Nilotinib and Imatinib	CMR at 12 months: 13/104 (Nilotinib) vs. 6/103 (Imatinib)	CML	0.208
NCT01061177	IV	Evaluate Nilotinib efficacy in newly diagnosed Ph^+^ CML	MR4.0 at 18 months: 38.3%; MMR: 56.2% (12 months), 61.1% (24 months)	CML	0.190
NCT00980018	IV	Assess low-grade AEs upon switching from Imatinib to Nilotinib	AE reduction after switching from Imatinib to Nilotinib: 71.2% (cycle 1), 82.7% (cycle 2), 84.6% (cycle 3)	CML	0.173
NCT01227577	IV	Evaluate deep molecular responses with Nilotinib over 4 years	Complete molecular response at 4 years: 34/128	CML	0.211
NCT01043874	IV	Assess MMR in Ph^+^ CML-CP patients with suboptimal response to Imatinib	MMR at 12 months: 51.1%	CML	0.156
NCT02546674	IV	Evaluate MR4.5 after 24 months Nilotinib in newly diagnosed CML	MR4.5 at 24 months: 35.3% (n=156)	CML	0.222
NCT01735955	IV	Long-term follow-up of Nilotinib-treated patients in extension study	Long-term clinical benefit maintained in 26/33 patients at week 192; 4 patients remained on treatment at week 528	CML, ALL, GIST, KIT melanoma	0.298

**Table 3.** Summary of clinical trials evaluating Nilotinib in Gastrointestinal Stromal Tumors (GIST). Phase II and III clinical trials investigating the use of Nilotinib in patients with GIST, particularly in those pretreated or refractory to standard therapies. The summarized endpoints include safety profiles, disease control duration, and progression-free survival. Notably, the incidence of SAEs appears higher in GIST trials compared to CML settings.

Clinical Trial	Phase	Aim	Result	Cancer Type	SAE
NCT01863745	II	Evaluate long-term safety of nilotinib	Safety monitoring: assessed SAEs and AEs in GIST patients	GIST	0.6
NCT01289028	III	Evaluate nilotinib efficacy in pretreated metastatic GIST	Stable disease for ≥4 months in 48.8% of GIST patients	GIST	0.48
NCT00471328	III	Compare nilotinib vs current treatments in GIST post- 1st/2nd line failure	Median progression-free survival (PFS) in GIST: 109 days	GIST	0.491

**Table 4.** Adverse events associated with Imatinib and Nilotinib treatment in clinical trial NCT02272777. The table summarizes the frequency and percentage of patients experiencing specific laboratory abnormalities in the Imatinib (n=112) and Nilotinib (n=113) treatment arms. Nilotinib-treated patients showed a higher incidence of elevated blood bilirubin, triglycerides, cholesterol, alanine aminotransferase, and low-density lipoprotein compared to those treated with Imatinib.

Adverse Events	Imatinib (n/N, %)	Nilotinib (n/N, %)
Blood bilirubin increased	2/112 (1.79%)	24/113 (21.24%)
Blood triglycerides increased	12/112 (10.71%)	17/113 (15.04%)
Blood cholesterol increased	3/112 (2.68%)	11/113 (9.73%)
Alanine aminotransferase increased	4/112 (3.57%)	9/113 (7.96%)
Blood glucose increased	9/112 (8.04%)	8/113 (7.08%)
Low density lipoprotein increased	1/112 (0.89%)	7/113 (6.19%)
Lipase increased	6/112 (5.36%)	7/113 (6.19%)

**Table 5.** Synergy analysis of Nilotinib and Paclitaxel in CTCL and AML cells. Combination Index (CI) values were calculated using CompuSyn software based on dose–response data in CTCL (Hut78) and AML (Molm13-luc) cells. Fa represents the fraction of cells affected at each total drug dose. CI values indicate the nature of interaction: <1 denotes synergy, =1 is additive, and >1 indicates antagonism. The combination exhibited strong to moderate synergy at intermediate doses and antagonism at very high doses in both cell types.

Total Dose (μM)	Fa (CTCL Hut78)	CI (CTCL Hut78)	Fa (AML Molm13-luc)	CI (AML Molm13- luc)
1.25	0.9999	510.744	0.9999	510.744
2.50	0.8167	1.45788	0.8167	1.45788
5.00	0.3276	0.57662	0.3276	0.57662
10.00	0.1872	0.65581	0.1872	0.65581
20.00	0.1072	0.87133	0.1072	0.87133
40.00	0.0738	1.31850	0.0738	1.31850

**Table 6.** Adverse events associated with Nilotinib and Paclitaxel combination therapy in solid tumor patients. Reported adverse events are from a clinical study involving 44 cancer patients treated with Nilotinib (300 mg BID) and Paclitaxel (80 mg/m^2^ weekly; 3 weeks on, 1 week off) which includes the most common all-grade events and grade ≥3 events or dose-limiting toxicities. Fatigue, neutropenia, and peripheral neuropathy were among the most frequently reported toxicities. Data adapted from Shin *et al*., [3,8].

Common Adverse Events (All Grades)	Grade ≥3 Adverse Events / Dose- Limiting Toxicities
Fatigue (52%)	Neutropenia (23%)
Peripheral neuropathy (45%)	Fatigue (11%)
Nausea (43%)	Hypertension (9%)
Diarrhea (34%)	Peripheral neuropathy (7%)
Alopecia (34%)	Hypophosphatemia (5%)
Vomiting (30%)	Febrile neutropenia (5%)
Anorexia (27%)	
Neutropenia (27%)	

## Data Availability

For original results such as public dataset analysis, cell viability assays and other analysis, please contact Xu Hannah Zhang PhD xuzhang@coh.org. FDA drug library screening data, please contact Sangkil Nam (snam@coh.org). For single cell analysis, please contact Xiwei Wu (xwu@coh.org); For original Mass spectrometry data please contact Roger Moore (rgmoore@coh.org). Other raw data may be found in a data supplement available with the online version of this article.

## Abbreviation:

CTCL: Cutaneous T Cell Lymphoma
CML: Chronic Myeloid Leukemia
Ph^+^ / Ph^−^: Philadelphia chromosome positive/negative
FDA: Food and Drug Administration
MAPK: Mitogen-Activated Protein Kinase
IC_50_: Half-Maximal Inhibitory Concentration
TKI: Tyrosine Kinase Inhibitor
PBMC: Peripheral Blood Mononuclear Cell
EMT: Epithelial-to-Mesenchymal Transition
LBS: Lipid Binding Site
AAD: All-Around Docking
MMR: Major Molecular Response
MR4.0/MR4.5: Deep/Very Deep Molecular Response - (4-log)/(4.5-log) reduction
SAE: Serious Adverse Event
AE: Adverse Event
ROR: Reporting Odds Ratio
NIH: National Institutes of Health
GIST: Gastrointestinal Stromal Tumor
CSC: Cancer Stem Cell
TGF-β: Transforming Growth Factor Beta
SMAD: SMAD is a gene family and protein domain name, derived from *C. elegans* Sma and *Drosophila* Mad
ENESTnd: Evaluating Nilotinib Efficacy and Safety in Clinical Trials—Newly Diagnosed
ECOG-ACRIN: Eastern Cooperative Oncology Group - American College of Radiology Imaging Network
NCI: National Cancer Institute
NCT: National Clinical Trial (identifier from ClinicalTrials.gov)
