# Supplementary material for: Repurposing Nilotinib as a Selective P38β Inhibitor in Hematopoietic Malignancies: Clinical Evidence and Mechanistic Insights
Source: J Clin Haematol. Author manuscript; Available in PMC 2026 Oct 8. (PMC13645396; doi:10.33696/haematology.6.067)
Supplement: Supplementary file 1 [file NIHMS2209924-supplement-Suppl__materials.pdf]

Suppl. Figure S1

Genes and Pathways Correlated with P38 isoforms in 11 CTCL LCT patients

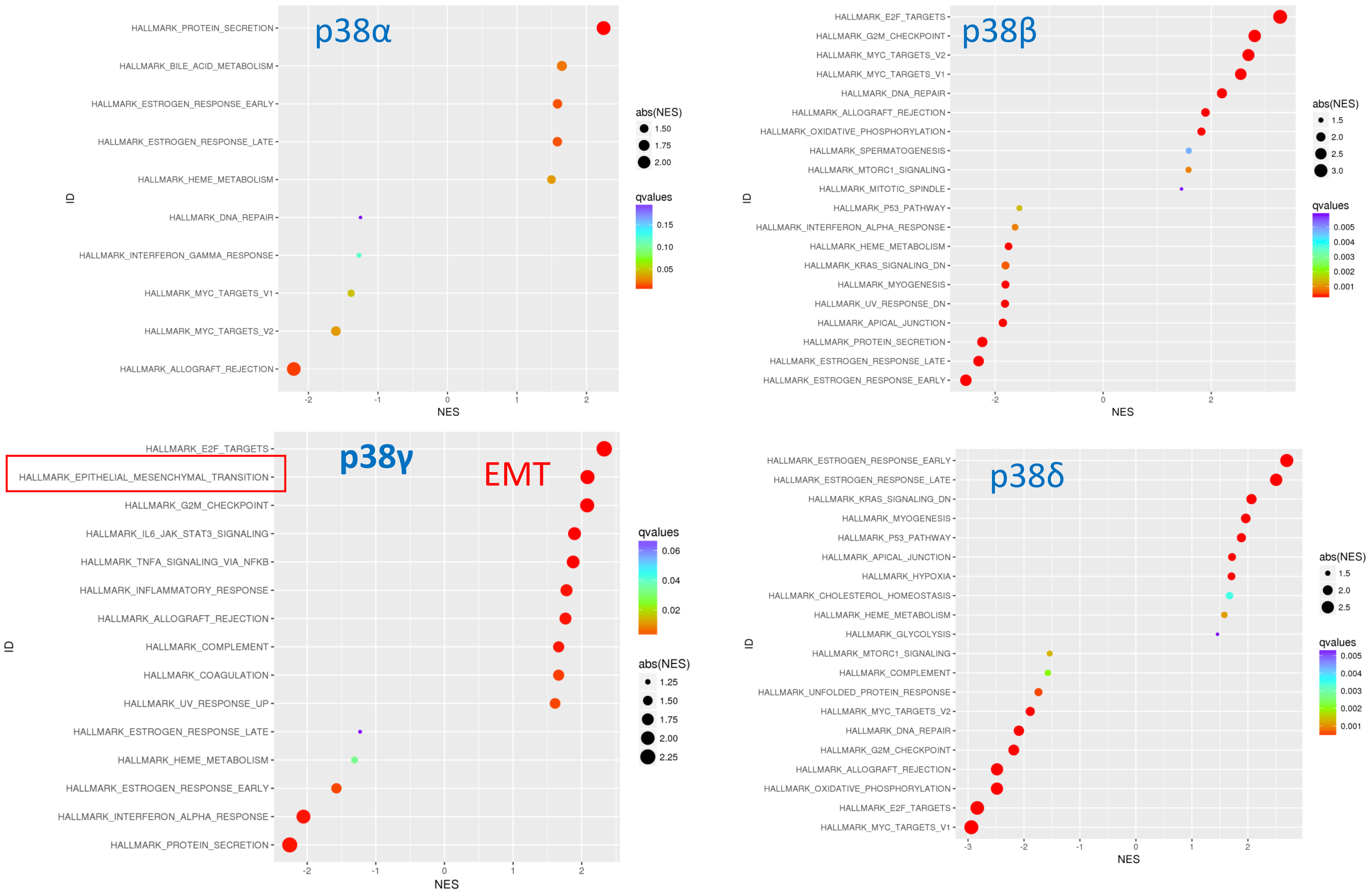

Suppl. Figure S2

A.

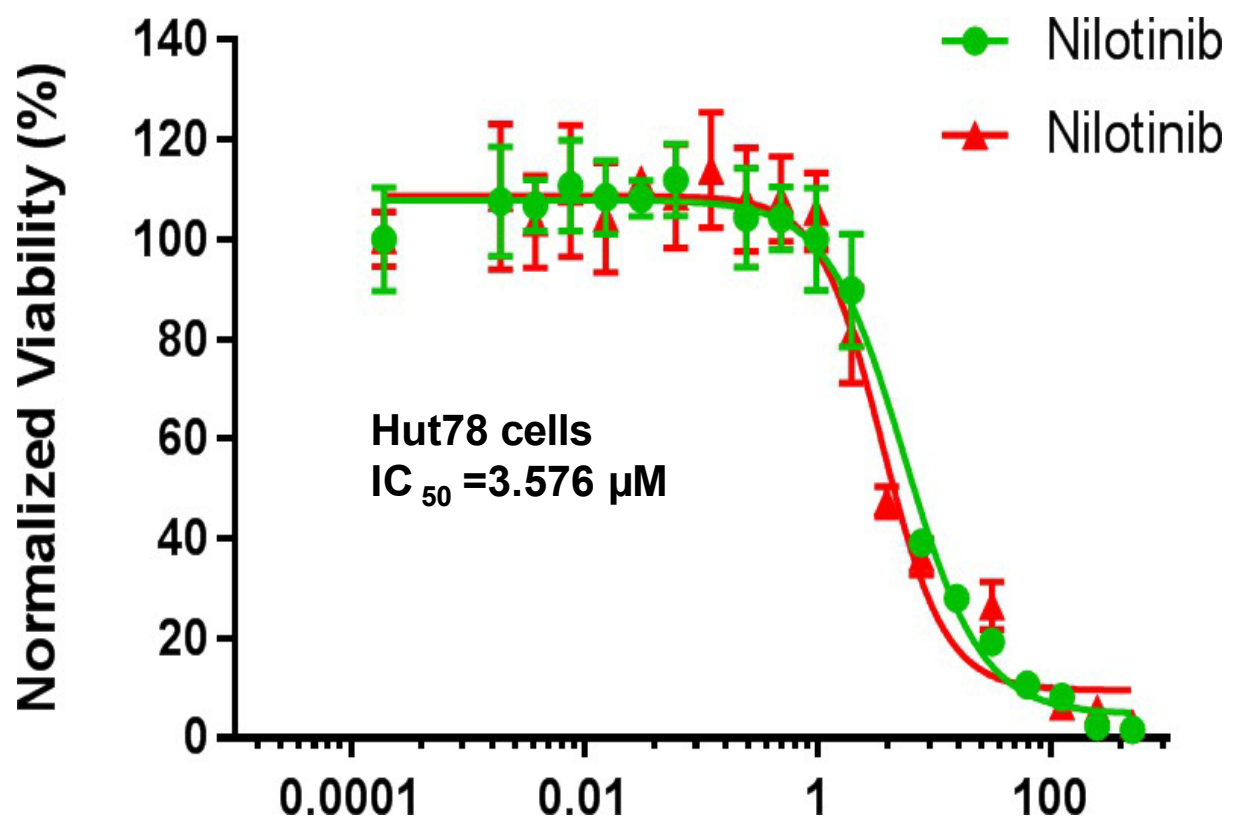

B.

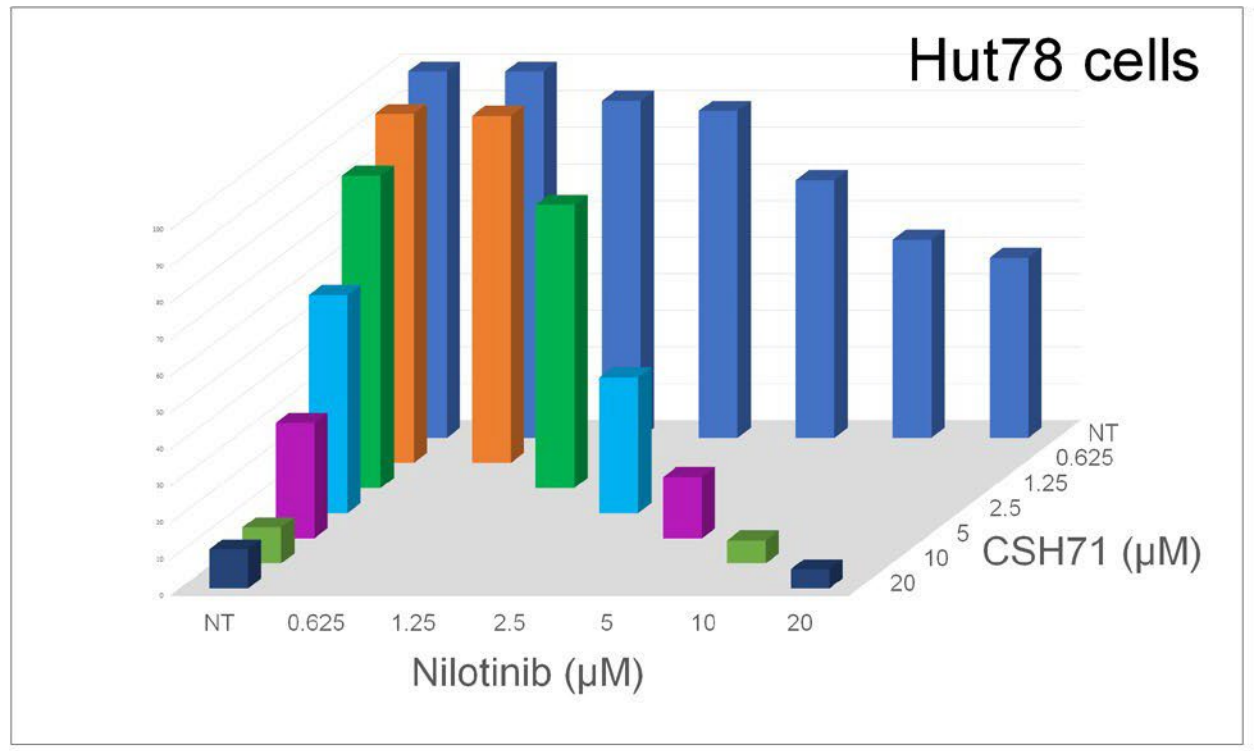

C.

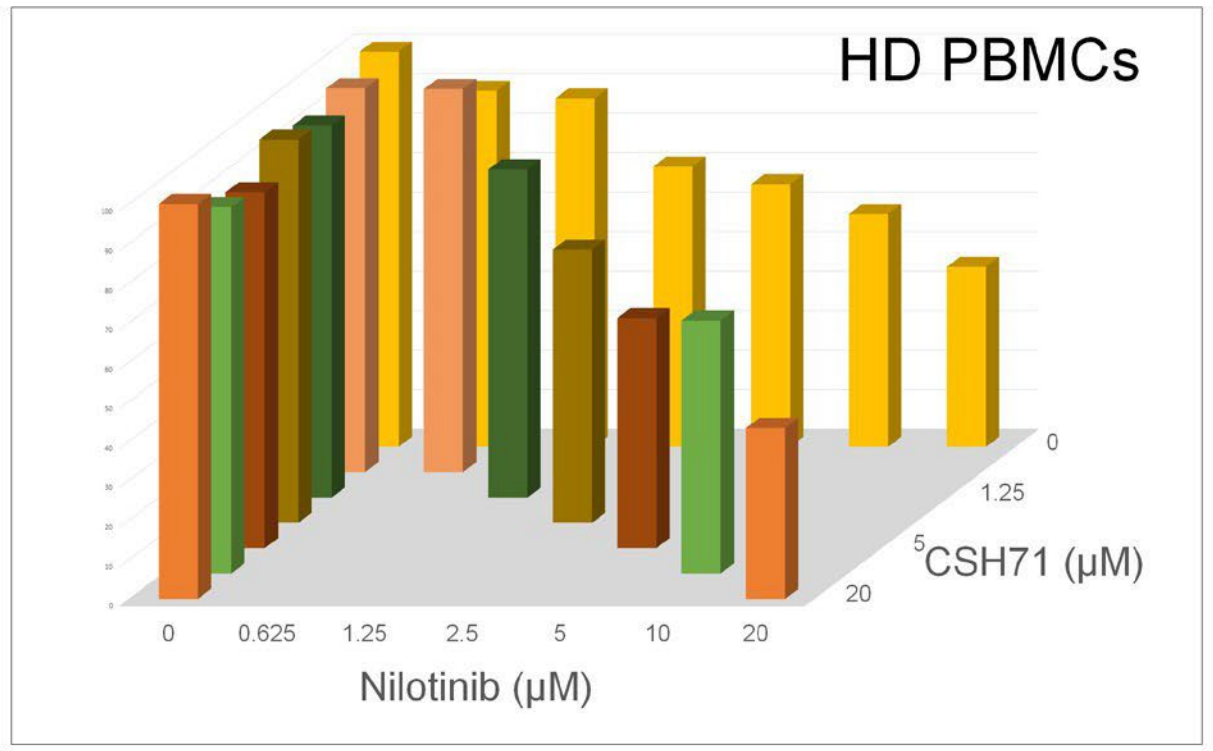

Hut78 cells

CSH71 (µM)	Nilotinib (µM)	Effects	CI
0.625	0.625	0.9473	1.2925
1.25	1.25	0.7729	0.9637
2.5	2.5	0.3704	0.69195
5	5	0.1607	0.74579
10	10	0.063	0.80215
20	20	0.0522	1.43398

Healthy donor's PBMCs (HD_PBMCs)

CSH71 (µM)	Nilotinib (µM)	Effects	CI
0.625	0.625	0.9701	6.25288
1.25	1.25	0.8309	3.9501
2.5	2.5	0.6917	13.4046
5	5	0.5825	40.3692
10	10	0.6406	65.1397
20	20	0.4328	278.329

Suppl. Figure S3

F7, p38γ kinase specific inhibitor, has synergistical effects with Nilotinib on Hut78 cells in a higher dosage (above 100nM)

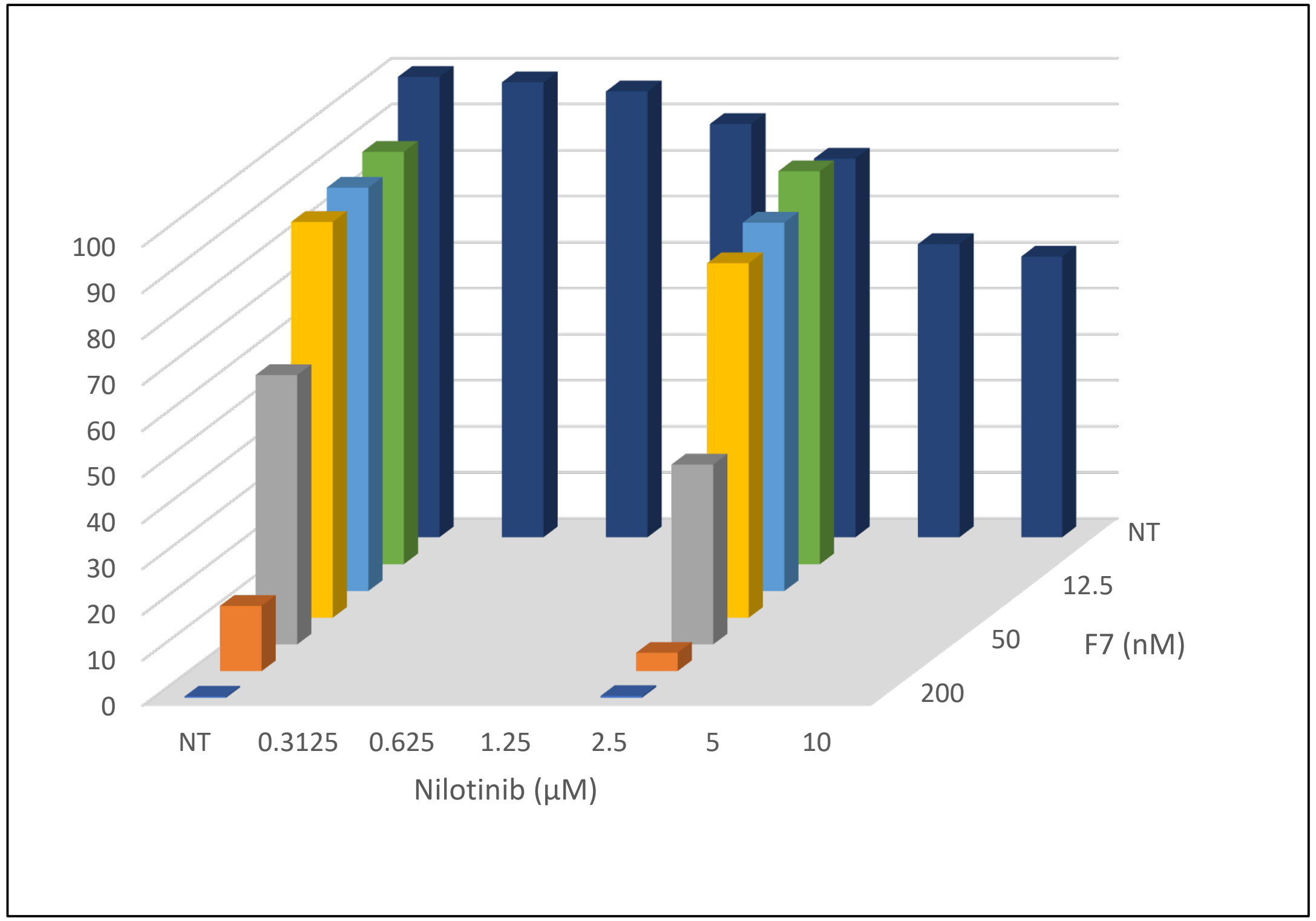

CI Data for Non-Constant Combo: F7NilC (F7+Nilo)

Dose F7	Dose Nilo	Effect	CI
6.25	2.5	0.8535	1.51367
12.5	2.5	0.8005	1.52995
25.0	2.5	0.7708	2.04989
50.0	2.5	0.3919	1.44989
100.0	2.5	0.0402	0.74045
200.0	2.5	0.0041	0.49740

F7+Nilotinib (Constant)
F7 (6.25-200nM)
Nilotinib (2.5µM)
-> Synergy at 100 and 200nM F7

Suppl. Figure S4

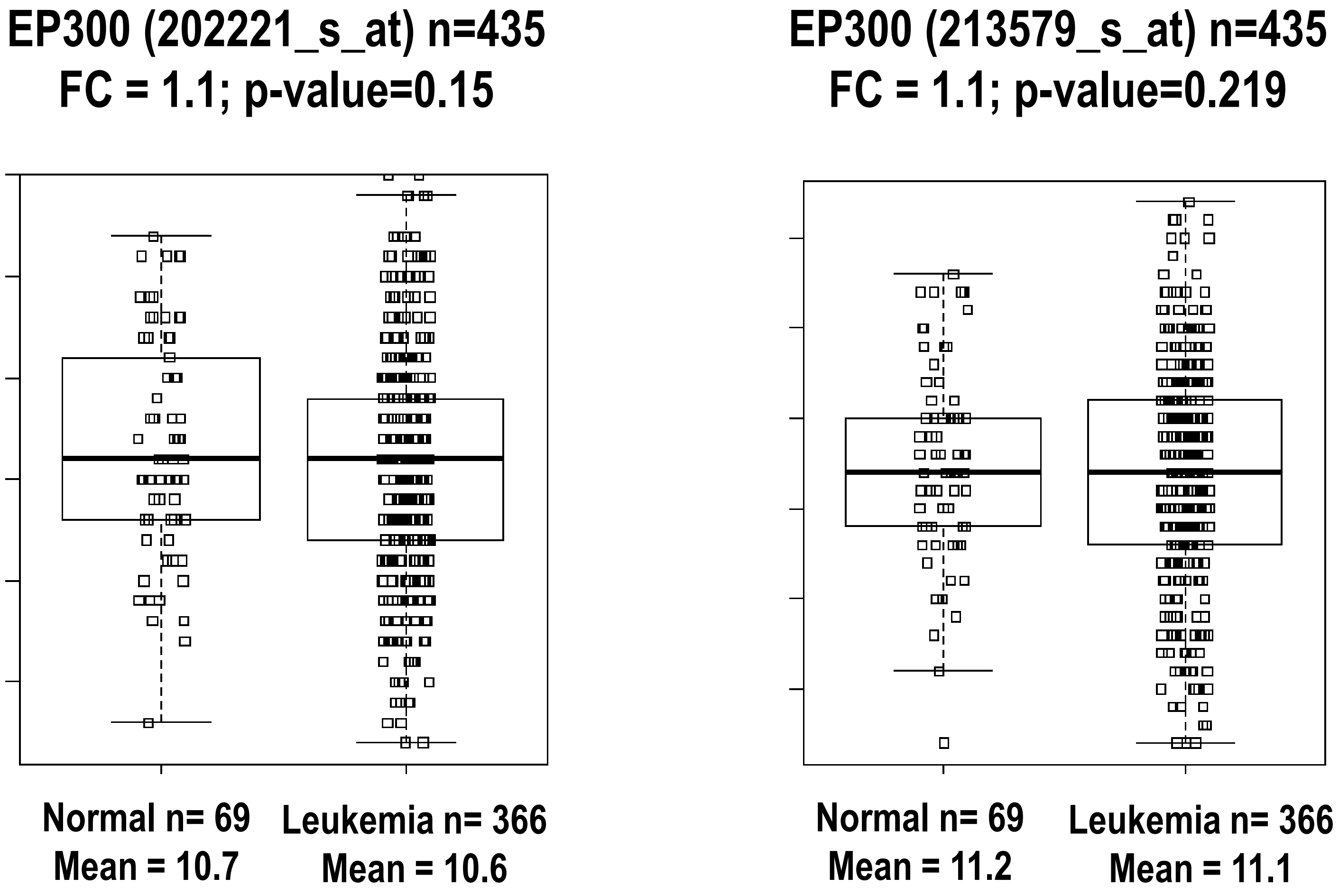

Suppl. Figure S5

Nilotinib and Paclitaxel Exhibit Synergistic Cytotoxicity in Hut 78 Cells

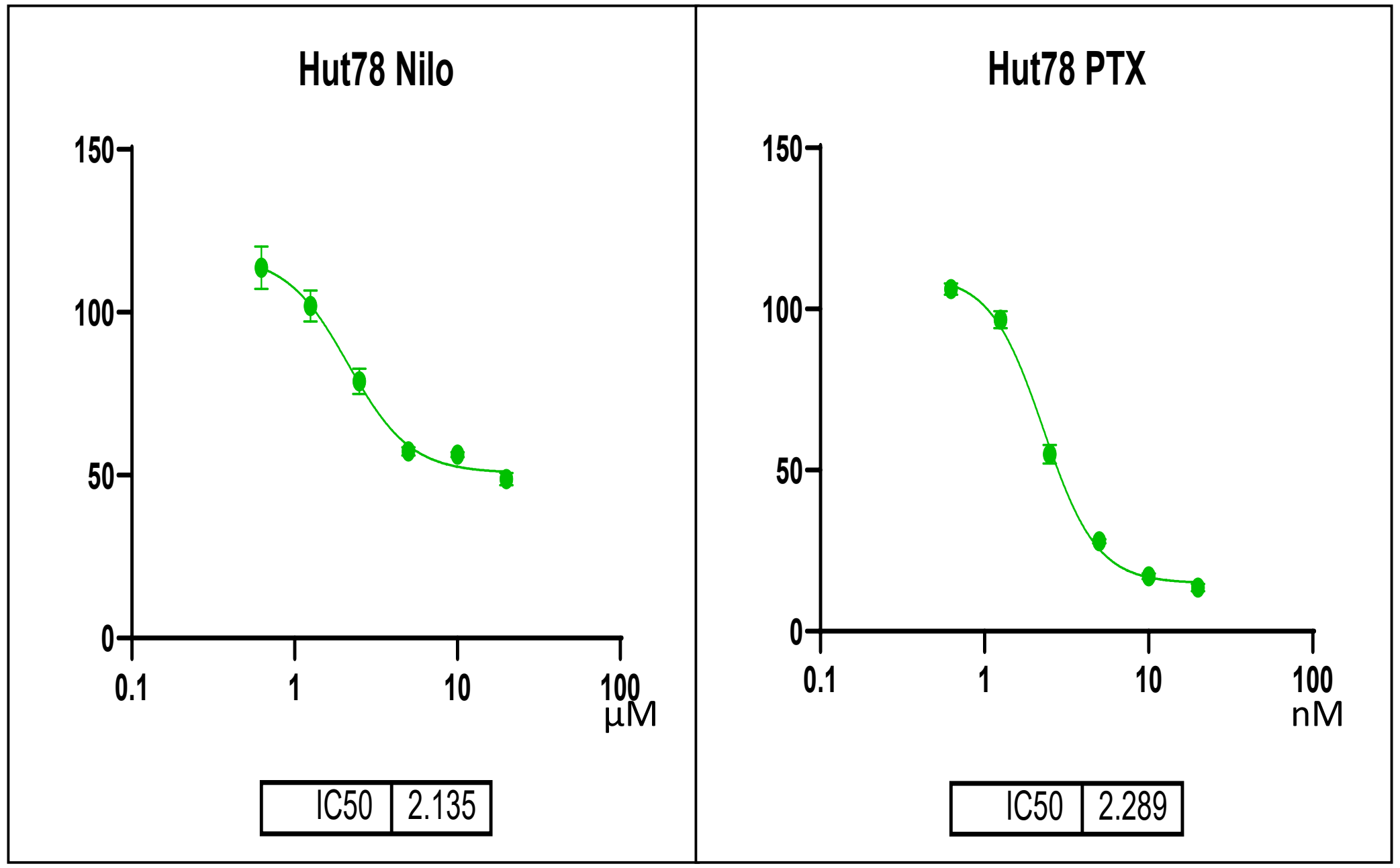

CI values		
Total Dose	Fa	CI Value
1.25	0.9999	510.744
2.5	0.8167	1.45788
5.0	0.3276	0.57662
10.0	0.1787	0.65581
20.0	0.1072	0.87133
40.0	0.0738	1.3185

Suppl. Figure S6

Nilotinib and Paclitaxel Exhibit Synergistic Cytotoxicity in AML Molm13-luc Cells

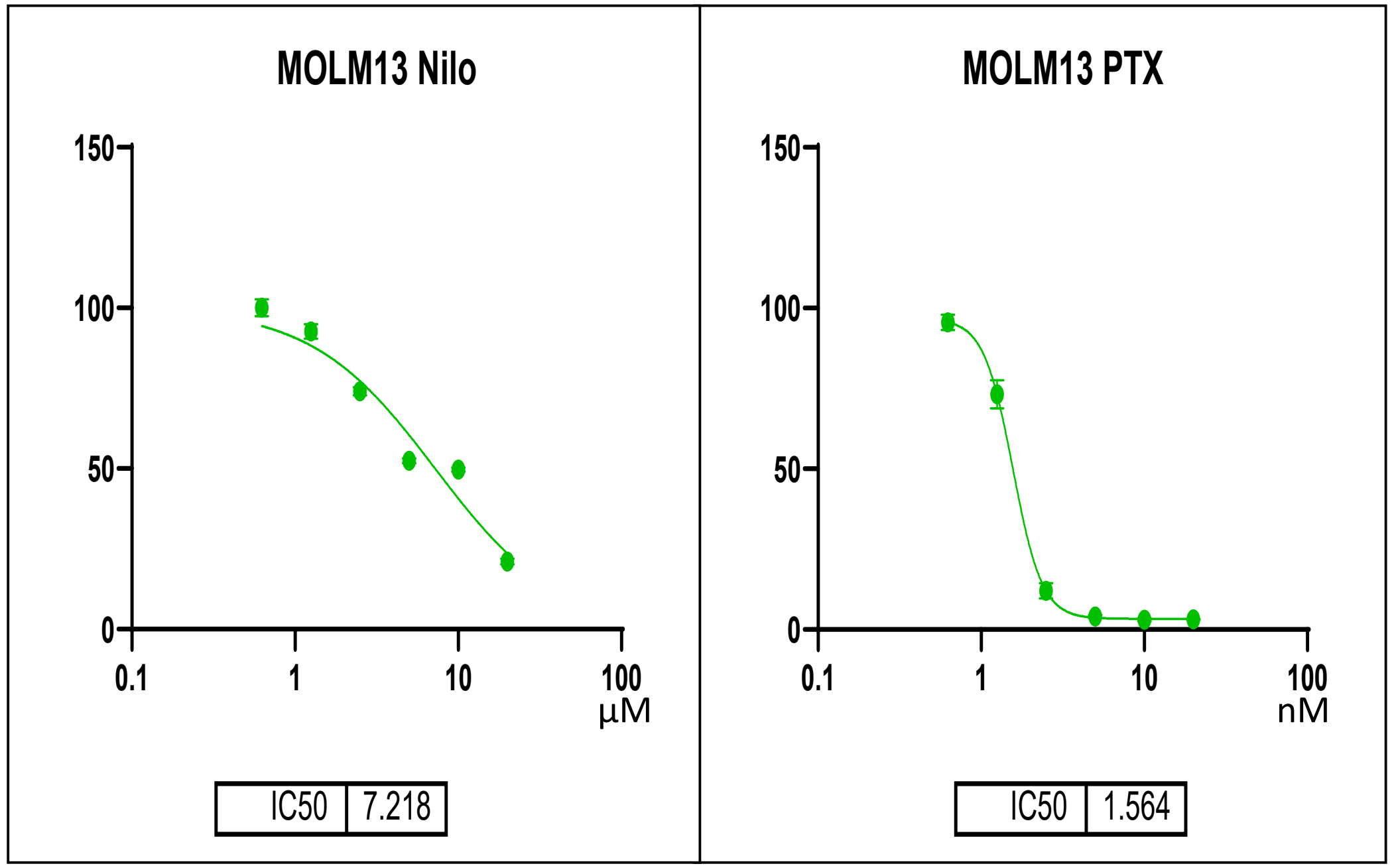

CI values		
Total Dose	Fa	CI Value
1.25	0.9675	3.37489
2.5	0.6611	1.54353
5.0	0.0858	0.61417
10.0	0.0398	0.80024
20.0	0.0323	1.4294
40.0	0.0215	2.29974

Supplementary Figure Legends:

Figure S1. Correlation of p38 isoforms with EMT gene signature in CTCL-LCT patient samples.

We analyzed RNA-seq dataset [GSE113113] which has 47 CTCL patients, including 11 with large cell transformation (LCT) and assess the relationship between p38 isoform expression and epithelial-to-mesenchymal transition (EMT). **Hallmark pathway** analysis revealed that only p38γ (MAPK12) expression was positively associated with EMT activation in LCT cases, whereas p38α, p38β, and p38δ showed no such correlation. These findings highlight a unique role for p38γ in promoting EMT in CTCL-LCT, supporting its contribution to disease aggressiveness.

Figure S2. Repurposing nilotinib in CTCL requires synergistic p38γ inhibition.

A. Nilotinib is toxic to the Hut78 cells. Nilotinib shown to the Hut78 cells with an IC50=3.576 μM. Data were normalized to the control value. Two representative experiments are presented, both of which are highly repeatable.
B. CSH71 is synergistic with Nilotinib in Hut 78 cells. Hut78 cells are treated with Nilotinib, CSH71 respectively with increasing dosages, and CSH71+Nilotinib combined treatments. The synergistic effects are found of CSH71 with Nilotinib in Hut78 cells with CI values 0.692, 0.746 and 0.802 respectively when the total (combined) doses are 5, 10 and 20 μM respectively.
C. No synergistic effects of Nilotinib with CSH71 in Healthy PBMCs. Healthy PBMCs are resistant to the CSH71, Nilotinib and combo drugs treatment in comparison to Hut78 cells. CI values are 13.4, 40,37 and 65.13 when the total (combined) doses are 5, 10 and 20 μM respectively.

Figure S3. Additive effects of Nilotinib with F7

F7, p38γ kinase inhibitor among p38 isoforms, has additive effects with Nilotinib in most of the range of concentration and synergistical effects on Hut78 cells only in a higher dosage (above 100nM).

Figure S4. RNA seq analysis of EP300 in cancer patients vs healthy normal controls

Public database analysis for RNA-Seq in leukemia samples (Mills et al 2009, GSE15061) showed that the expression level of epigenetic regulator EP300, a known tumor suppressor, shows no significant change compared to non-cancerous samples.

Figure S5. Combination index (CI) analysis of Nilotinib and Paclitaxel in CTCL Hut78 cells.

CI values calculated using CompuSyn software revealed dose-dependent synergy between the two drugs, with strongest synergy observed at Fa = 0.3276 (CI = 0.5766) and sustained synergistic effects down to Fa = 0.1072 (CI = 0.8713). Mild antagonism was observed at the extremes of the dose range, but overall the combination demonstrated enhanced cytotoxicity, supporting its potential as a rational therapy in CTCL.

Figure S6. Combination index (CI) analysis of Nilotinib and Paclitaxel in AML Molm13-luc cells.

CI analysis using CompuSyn software revealed a defined window of dose-dependent synergy, with strong synergy at 5.0 μM (CI = 0.6142) and moderate synergy at 10.0 μM (CI = 0.8002). Antagonistic effects were observed at both lower and higher doses, indicating that optimal cytotoxic enhancement occurs within a narrow concentration range.
